# National Trends and Demographic Disparities in Mortality Involving Co-Recorded Parkinson’s Disease and Dementia in the United States, 1999–2025: A CDC WONDER Analysis

**DOI:** 10.3390/neurosci7030066

**Published:** 2026-06-10

**Authors:** Hassaan Abid, Sohana Memon, Vishan Das, Kaneez Fatima, Muhammad Mukhlis, Muhammad Vazaym

**Affiliations:** 1 Department of Internal Medicine, Indiana University School of Medicine, Indianapolis, IN 46202, USA; 2 Department of Internal Medicine, Liaquat University of Medical and Health Sciences (LUMHS), Jamshoro 76090, Pakistan; 23lm054@my.lumhs.edu.pk (V.D.); 24lm227@my.lumhs.edu.pk (M.V.); 3 Department of Internal Medicine, CMH Institute of Medical Science, Multan 53400, Pakistan; kaneezfatima7133@cims.pk; 4 Department of Internal Medicine, Ayub Medical College, Abbottabad 22020, Pakistan; 22-148@ayubmed.edu.pk

**Keywords:** Parkinson’s disease, dementia, neurodegenerative disorders, mortality trends, CDC WONDER

## Abstract

Background: Parkinson’s disease and dementia are major neurodegenerative disorders that substantially contribute to disability, dependency, and mortality worldwide. Although prior CDC WONDER studies have separately evaluated Parkinson’s disease and dementia mortality trends, fewer analyses have examined national mortality patterns in which both conditions are recorded on death certificates simultaneously over extended time periods. Methods: We analyzed U.S. death certificates from 1999 through 2025 using the CDC WONDER Multiple Cause of Death database, identifying deaths among adults aged ≥45 years in which both Parkinson’s disease (ICD-10 G20) and dementia-related codes (F01, F03, G30, G31) were recorded anywhere on the certificate. This operational definition captures co-recorded diagnoses and does not identify clinically confirmed Parkinson’s disease dementia. Age-adjusted mortality rates (AAMRs) per 100,000 were standardized to the 2000 U.S. standard population, a method that controls for shifts in population age structure over time and allows valid temporal comparisons independent of absolute population growth. Joinpoint regression was used to quantify trends. Sensitivity analyses excluded 2025 provisional data and the COVID-19 period (1999–2019). Results: A total of 337,721 deaths were identified. Overall AAMR increased from 5.75 (95% CI: 5.60–5.90) in 1999 to 11.15 (95% CI: 10.98–11.32) in 2025 (AAPC: 2.07; *p* = 0.002). A sharp transient increase occurred in 2020, attributable to pandemic-related factors including disproportionate COVID-19 mortality among older adults with neurodegenerative conditions, care disruptions, and changes in death-certificate coding practices. Following this pandemic-era peak, AAMRs declined significantly through 2025 and should be interpreted cautiously given provisional data. Males (AAPC: 2.14), non-Hispanic White individuals (AAPC: 2.29), the Midwest region (AAPC: 2.65), and non-metropolitan areas carried the highest mortality burden. Mortality was greatest among adults aged ≥85 years. Conclusion: Population-level death rates involving co-recorded Parkinson’s disease and dementia demonstrated significant temporal changes over the study period, with marked demographic and geographic disparities. These findings reflect death-certificate surveillance data and cannot establish clinical co-occurrence, causal relationships, or individual disease risk.

## 1. Introduction

Neurodegenerative disorders are becoming a more common cause of death and disability around the world. Their burden is growing along with the aging of the global population. Dementia affects tens of millions of people around the world and is still one of the main causes of death and dependency in older adults [1]. Parkinson’s disease (PD) is another major neurodegenerative disease that affects almost 6 million people around the world. Its prevalence is expected to rise significantly in the next few decades as the population ages [2]. Dementia-related diagnoses are frequently recorded among individuals with Parkinson’s disease, particularly in advanced neurodegenerative illness, and are associated with greater disability, institutionalization, and mortality burden [3]. In the United States, neurological conditions account for a substantial and growing proportion of mortality, with both PD and dementia contributing significantly to this trend [4].

Parkinson’s disease is a progressive neurodegenerative disorder characterized primarily by motor symptoms such as bradykinesia, rigidity, and tremor, alongside a wide range of non-motor manifestations. Among these, cognitive decline and dementia represent some of the most clinically significant complications. Parkinson’s disease dementia reflects advanced neurodegenerative involvement affecting cortical and subcortical networks, often associated with α-synuclein pathology and overlapping Alzheimer-type changes [5]. Clinically, the presence of dementia in PD is associated with more rapid functional decline, greater care dependence, increased institutionalization, and higher mortality compared with Parkinson’s disease without dementia [6]. As life expectancy continues to increase, the coexistence of PD and dementia has become an increasingly relevant concern for patients, caregivers, and healthcare systems.

The existing literature has demonstrated rising mortality associated with both Parkinson’s disease and dementia, along with important disparities across demographic groups. Higher mortality rates are generally observed among older adults, males, and certain racial and geographic populations, reflecting differences in disease burden, healthcare access, and diagnostic patterns. Similarly, dementia-related mortality varies substantially by age, sex, and race, with disproportionately higher burden observed among older populations and some minority groups [2,7]. However, nearly all prior studies have primarily focused on incidence and demographic variation rather than mortality outcomes. As a result, mortality associated with co-recorded conditions remains insufficiently characterized. Although prior CDC WONDER studies have separately evaluated Parkinson’s disease and dementia mortality trends, fewer analyses have examined national mortality patterns in which both conditions are recorded on death certificates simultaneously over extended time periods.

This study examines temporal trends in mortality involving Parkinson’s disease and dementia-related diagnoses among adults aged 45 years and older in the United States using national mortality data from the CDC WONDER Multiple Cause of Death database. The analysis was intended to evaluate mortality patterns involving co-recorded Parkinson’s disease and dementia-related diagnoses rather than clinically adjudicated Parkinson’s disease dementia. By analyzing age-adjusted death rates over more than two decades, this study aims to provide a comprehensive assessment of the evolving mortality burden associated with these co-recorded neurodegenerative conditions.

## 2. Methods

### 2.1. Data Source

In this descriptive, population-based retrospective study, mortality data were obtained from the Centers for Disease Control and Prevention Wide-ranging Online Data for Epidemiologic Research (CDC WONDER) Multiple Cause of Death database [8]. This study analyzed deaths among U.S. residents aged 45 years and older from 1999 through 2025. The analysis evaluated deaths in which both Parkinson’s disease and dementia ICD-10 codes were recorded whether present as underlying or contributing causes of death. Parkinson’s disease was identified using ICD-10 code G20. Dementia-related diagnoses were identified using ICD-10 codes F01 (vascular dementia), F03 (unspecified dementia), G30 (Alzheimer disease), and G31 (other degenerative diseases of the nervous system). Previous published studies have used similar ICD-10 codes for Parkinson’s disease and dementia [9,10]. This study did not identify clinically confirmed Parkinson’s disease dementia (PDD) or dementia with Lewy bodies. Instead, the analysis captured deaths in which Parkinson’s disease (ICD-10 G20) and selected dementia-related ICD-10 codes were recorded anywhere on the death certificate. These dementia codes included vascular dementia (F01), unspecified dementia (F03), Alzheimer disease (G30), and other degenerative nervous system diseases (G31). Accordingly, the dataset reflects heterogeneous co-recorded neurodegenerative conditions rather than a specific clinical diagnosis. Death-certificate data cannot determine disease onset, diagnostic timing, disease severity, causal relationships, or whether dementia developed before or after Parkinson’s disease. The Multiple Cause of Death Public Use Record was used to identify deaths where Parkinson’s disease and dementia-related codes appeared anywhere on the death certificate regardless of the underlying cause of death. This study used publicly available de-identified data and therefore did not require institutional review board approval. Reporting followed the Strengthening the Reporting of Observational Studies in Epidemiology (STROBE) recommendations [11].

### 2.2. Data Extraction

Data were extracted from the Centers for Disease Control and Prevention Wide-ranging Online Data for Epidemiologic Research (CDC WONDER) Multiple Cause of Death (MCD) database, covering all 50 U.S. states and the District of Columbia from 1999 to 2025. The dataset included annual mortality counts, population denominators, year and geographic location of death, and available demographic characteristics. Additional variables included age at death, sex, race/ethnicity, U.S. Census region, urbanization level, and underlying and contributing causes of death as recorded on death certificates. Age was categorized into the following strata for neurodegenerative mortality analyses: 45–64 years, 65–74 years, 75–84 years, and ≥85 years. Sex was classified as male or female as reported on death certificates. Race and ethnicity were categorized as non-Hispanic White, non-Hispanic Black or African American, non-Hispanic American Indian or Alaska Native, non-Hispanic Asian or Pacific Islander, and Hispanic or Latino, in accordance with CDC WONDER classifications. Geographic distribution was assessed using U.S. Census regions (Northeast, Midwest, South, and West), with additional state-level analyses performed where applicable. Urbanization status was defined according to the National Center for Health Statistics (NCHS) urban–rural classification scheme and categorized as metropolitan and nonmetropolitan areas.

Urbanization analyses were limited to 1999–2020 because NCHS urban–rural classification data were unavailable beyond 2020.

### 2.3. Statistical Analysis

Age-adjusted mortality rates (AAMRs) per 100,000 population were calculated for adults aged ≥45 years using CDC WONDER mortality data from 1999 through 2025. Age-adjusted mortality rates were standardized to the 2000 U.S. standard population in accordance with CDC and National Center for Health Statistics recommendations for longitudinal mortality trend analyses. This method allows comparison of rates over time while minimizing the influence of changing population age structure. Although the absolute U.S. population increased substantially during the study period, age-adjusted rates represent standardized population estimates rather than raw mortality counts. Temporal trends were evaluated using Joinpoint regression analysis [12]. Joinpoint models were fit using log-linear regression with weighted least squares based on standard errors of the age-adjusted mortality rates. A maximum of five joinpoints was permitted, with model selection determined using Monte Carlo permutation testing. Minimum segment length requirements followed National Cancer Institute Joinpoint program defaults. Annual percent change (APC) and average annual percent change (AAPC) with corresponding 95% confidence intervals (CIs) were calculated to quantify mortality trends over time. Statistical significance was defined as a two-sided *p* < 0.05. All analyses were performed using the Joinpoint Regression Program (version 5.4). The analysis period was extended to 2025 using the latest CDC WONDER mortality release to provide an updated assessment of long-term trends. Sensitivity analyses were performed for 1999–2024 to exclude provisional mortality statistics from 2025 and for 1999–2019 to avoid the COVID-19 timeframe. Further analyses were conducted by restricting the dataset to deaths where dementia was listed as the underlying cause and, separately, to deaths where Parkinson’s disease was listed as the underlying cause in order to determine whether inclusion of contributing causes of death influenced the identified temporal trends. Using the same analytical framework, joinpoint regression was performed again to compare temporal patterns with the initial analysis focused on mortality related to dementia and Parkinson’s disease.

## 3. Results

### 3.1. Annual AAMR Trends for Parkinson’s and Dementia

**Figure 1:**
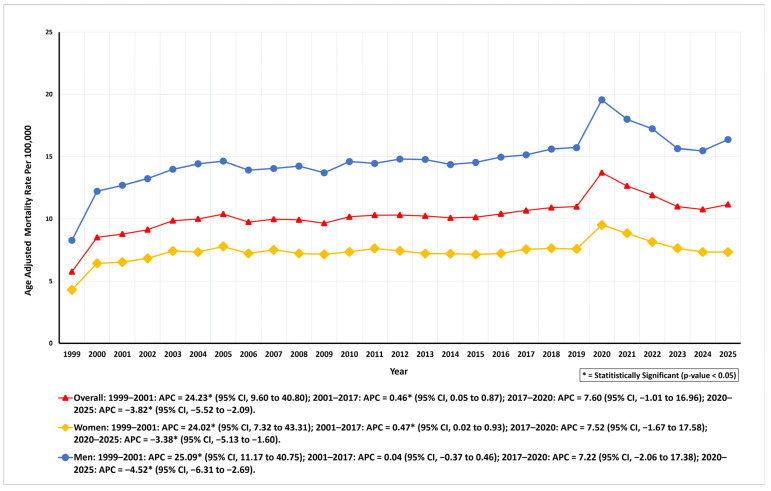
Overall and Sex-stratified Age-adjusted mortality rates (per 100,000) for Co-Recorded Parkinson’s disease- and Dementia-related mortality among U.S. adults aged ≥45, 1999 to 2025.

Out of a total of 337,721 deaths, males accounted for the majority (56.51%), while females represented a smaller proportion (43.49%). Overall AAMRs increased from 5.75 (95% CI: 5.60–5.90) in 1999 to 11.15 (95% CI: 10.98–11.32) in 2025 (AAPC: 2.07; 95% CI: 0.73–3.42, *p* = 0.002). AAMR increased significantly from 1999 to 2001 (APC: 24.23; 95% CI: 9.60 to 40.80, *p* = 0.002), and continued to rise significantly from 2001 to 2017 (APC: 0.46; 95% CI: 0.05 to 0.87, *p* = 0.030). From 2017 to 2020, AAMR demonstrated a non-significant increase, followed by a significant decline from 2020 to 2025 (APC: -3.82; 95% CI: −5.52 to −2.09, *p* = 0.0003) (Figure 1) (Supplemental Tables S1 and S3).

### 3.2. Age-Stratified AAMR Trends for Parkinson’s and Dementia

The highest average crude mortality rate was observed among individuals aged ≥85 years (93.21), followed by those aged 75–84 years (37.99), 65–74 years (5.79), and 45–64 years (0.24).

Among individuals aged 45–64 years, crude mortality rates (CMR) increased from 0.09 (95% CI: 0.07–0.12) in 1999 to 0.33 (95% CI: 0.29–0.37) in 2025 (AAPC: 3.55; 95% CI: 0.33–6.88, *p* = 0.031). Mortality rates increased significantly from 1999 to 2018 (APC: 4.37; 95% CI: 3.13–5.63, *p* < 0.000001). From 2018 to 2025, rates remained stable.

In individuals aged 65–74 years, CMRs increased from 3.34 (95% CI: 3.08–3.61) in 1999 to 6.98 (95% CI: 6.70–7.25) in 2025 (AAPC: 2.65; 95% CI: 1.01–4.31, *p* = 0.001). Mortality rates increased significantly from 1999 to 2001 (APC: 22.76; 95% CI: 1.47–48.52, *p* = 0.036), remained relatively stable from 2001 to 2015, and then increased significantly from 2015 to 2020 (APC: 7.72; 95% CI: 3.80–11.80, *p* = 0.001). From 2020 to 2025, mortality rates declined significantly (APC: −2.58; 95% CI: −4.77 to −0.34, *p* = 0.027).

**Figure 2:**
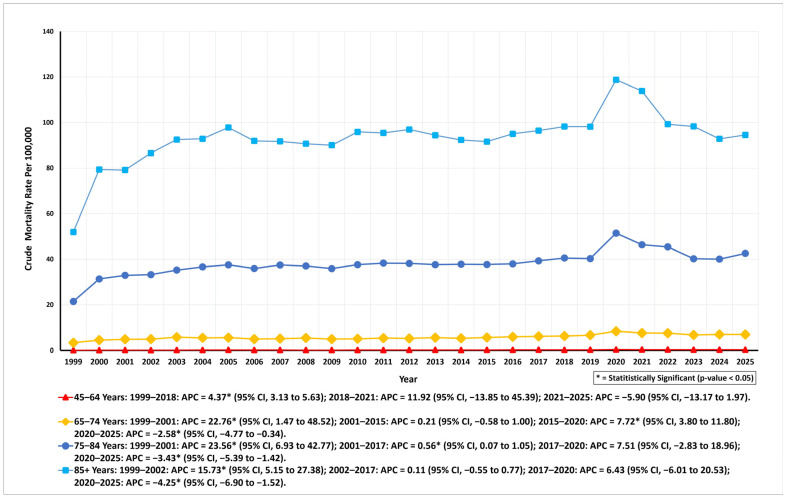
Age-stratified age-adjusted mortality rates per 100,000 Co-Recorded Parkinson’s disease- and Dementia-related mortality in United States adults aged ≥45 years, 1999–2025.

Among individuals aged 75–84 years, CMRs increased from 21.47 (95% CI: 20.65–22.29) in 1999 to 42.54 (95% CI: 41.62–43.47) in 2025 (AAPC: 2.16; 95% CI: 0.58–3.76, *p* = 0.007). Mortality rates increased significantly from 1999 to 2001 (APC: 23.56; 95% CI: 6.93–42.77, *p* = 0.007) and continued to rise gradually from 2001 to 2017 (APC: 0.56; 95% CI: 0.07–1.05, *p* = 0.029). Rates remained stable until 2020. From 2020 to 2025, mortality rates declined significantly (APC: −3.43; 95% CI: −5.39 to −1.42, *p* = 0.002). Among individuals aged ≥85 years, CMRs increased from 51.93 (95% CI: 49.73–54.12) in 1999 to 94.57 (95% CI: 92.18–96.96) in 2025 (AAPC: 1.64; 95% CI: −0.15 to 3.47, *p* = 0.073). Mortality rates increased significantly from 1999 to 2002 (APC: 15.73; 95% CI: 5.15–27.38, *p* = 0.005), remained relatively stable from 2002 to 2020. From 2020 to 2025, mortality rates declined significantly (APC: −4.25; 95% CI: −6.90 to −1.52, *p* = 0.005) (Figure 2) (Supplemental Tables S2 and S4).

### 3.3. Race-Stratified AAMR Trends for Parkinson’s and Dementia

The White population had the highest average AAMR (11.16), followed by the Hispanic or Latino population (7.41), the Black or African American population (6.04), and the Asian or Pacific Islander population (5.53).

In the Hispanic or Latino population, AAMRs increased from 4.15 (95% CI: 3.48–4.82) in 1999 to 7.84 (95% CI: 7.37–8.33) in 2025 (AAPC: 1.74; 95% CI: 0.43–3.08, *p* = 0.009). AAMR rose significantly from 1999 to 2006 (APC: 6.78; 95% CI: 4.16–9.47, *p* < 0.001) and then remained relatively stable from 2006 to 2020. From 2020 to 2025, mortality rates declined significantly (APC: −5.53; 95% CI: −7.38 to −3.64, *p* < 0.001).

In the Asian or Pacific Islander population, AAMRs increased from 1.89 (95% CI: 1.27–2.70) in 1999 to 5.84 (95% CI: 5.32–6.38) in 2025 (AAPC: 3.17; 95% CI: −0.82 to 7.32, *p* = 0.121). AAMR showed a non-significant increase from 1999 to 2002 and then remained relatively stable from 2002 to 2025.

In the Black or African American population, AAMRs increased from 3.06 (95% CI: 2.65–3.46) in 1999 to 6.75 (95% CI: 6.31–7.21) in 2025 (AAPC: 2.90; 95% CI: 0.63–5.21, *p* = 0.012). AAMR showed a non-significant increase from 1999 to 2002, and then rose steadily from 2002 to 2025 (APC: 1.21; 95% CI: 0.61–1.81, *p* < 0.001).

**Figure 3:**
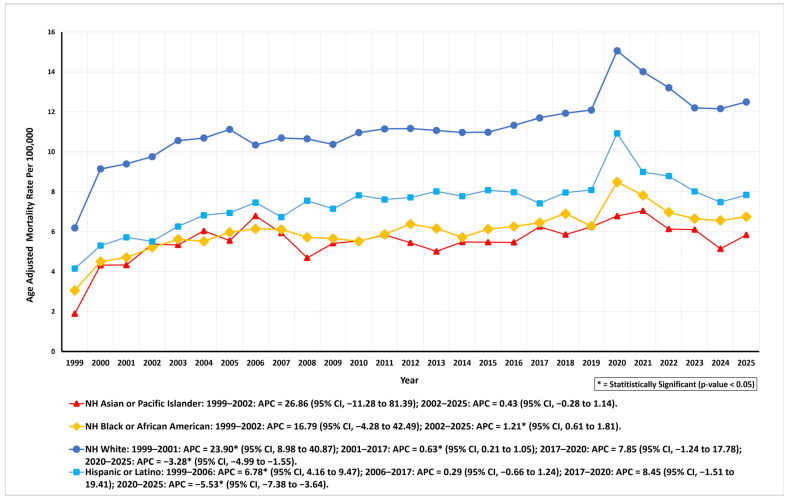
Race-stratified Age-adjusted mortality rates (per 100,000) for Co-Recorded Parkinson’s disease- and Dementia-related mortality among U.S. adults aged ≥45, 1999 to 2025.

In the White population, AAMRs increased from 6.19 (95% CI: 6.02–6.36) in 1999 to 12.50 (95% CI: 12.29–12.71) in 2025 (AAPC: 2.29; 95% CI: 0.90–3.70, *p* = 0.001). AAMR rose significantly from 1999 to 2001 (APC: 23.90; 95% CI: 8.98–40.87, *p* = 0.003) and then increased steadily from 2001 to 2017 (APC: 0.63; 95% CI: 0.21–1.05, *p* = 0.005). This was followed by a sharper, though non-significant, increase through 2020. From 2020 to 2025, mortality rates declined significantly (APC: −3.28; 95% CI: −4.99 to −1.55, *p* = 0.001) (Figure 3) (Supplemental Tables S2 and S5).

### 3.4. Urbanization-Stratified AAMR Trends for Parkinson’s and Dementia

Non-metropolitan areas had the higher average AAMR (10.45), followed by metropolitan areas (9.88). AAMRs in metropolitan areas increased from 5.81 (95% CI: 5.64–5.98) in 1999 to 13.35 (95% CI: 13.14–13.56) in 2020 (AAPC: 3.54; 95% CI: 2.35–4.74, *p* < 0.000001). The trend showed a significant increase from 1999 to 2001 (APC: 23.96; 95% CI: 10.96–38.48, *p* = 0.0009), and continued to rise from 2001 to 2018 (APC: 0.44; 95% CI: 0.13–0.76, *p* = 0.009), and then a sharper significant increase from 2018 to 2020 (APC: 11.95; 95% CI: 4.54–19.90, *p* = 0.003).

**Figure 4:**
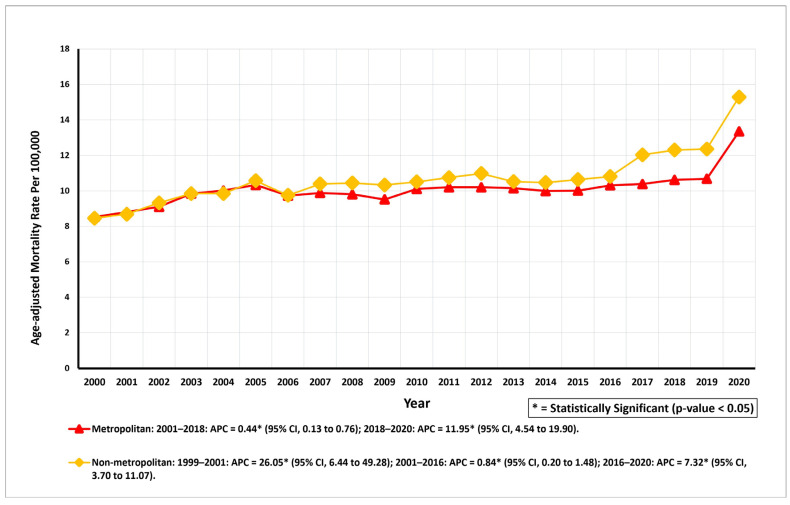
Urbanization-stratified age-adjusted mortality rates per 100,000 for Co-Recorded Parkinson’s disease- and Dementia-related mortality in United States adults aged ≥45 years, 1999–2020.

Similarly, in non-metropolitan areas, AAMRs increased from 5.62 (95% CI: 5.27–5.96) in 1999 to 15.29 (95% CI: 14.79–15.79) in 2020 (AAPC: 4.23; 95% CI: 2.54–5.96, *p* = 0.000001). The trend showed a significant increase from 1999 to 2001 (APC: 26.05; 95% CI: 6.44–49.28, *p* = 0.011), followed by a continued notable rise from 2001 to 2016 (APC: 0.84; 95% CI: 0.20–1.48, *p* = 0.013). Rates further showed a significant increase from 2016 to 2020 (APC: 7.32; 95% CI: 3.70–11.07, *p* = 0.0006) (Figure 4) (Supplemental Tables S2 and S6).

### 3.5. Census Region-Stratified AAMR Trends for Parkinson’s and Dementia

The highest average AAMR was observed in the Midwest region (11.52), followed by the South region (10.13), the West region (10.11), and the Northeast region (9.19).

In the Northeast region, AAMRs increased from 5.39 (95% CI: 5.07–5.70) in 1999 to 9.23 (95% CI: 8.88–9.59) in 2025 (AAPC: 1.53; 95% CI: 0.01–3.07, *p* = 0.048). AAMR rose rapidly from 1999 to 2001 (APC: 21.26; 95% CI: 5.98–38.74, *p* = 0.008) and then increased significantly from 2001 to 2017 (APC: 0.68; 95% CI: 0.21–1.15, *p* = 0.007). This was followed by a sharper, though non-significant, increase through 2020. From 2020 to 2025, mortality rates declined notably (APC: −5.66; 95% CI: −7.67 to −3.60, *p* < 0.001).

In the Midwest region, AAMRs increased from 6.12 (95% CI: 5.80–6.44) in 1999 to 12.54 (95% CI: 12.15–12.94) in 2025 (AAPC: 2.65; 95% CI: 0.44–4.90, *p* = 0.019). AAMR showed a non-significant increase from 1999 to 2001, and then rose steadily from 2001 to 2025 (APC: 0.87; 95% CI: 0.41–1.33, *p* < 0.001).

In the South region, AAMRs increased from 5.96 (95% CI: 5.69–6.22) in 1999 to 12.29 (95% CI: 12.01–12.59) in 2025 (AAPC: 2.34; 95% CI: 0.96–3.73, *p* < 0.001). AAMR rose significantly from 1999 to 2003 (APC: 9.54; 95% CI: 4.51–14.81, *p* < 0.001) and then showed a slower, non-significant increase from 2003 to 2020. From 2020 to 2025, mortality rates declined significantly (APC: −2.36; 95% CI: −4.26 to −0.42, *p* = 0.020).

**Figure 5:**
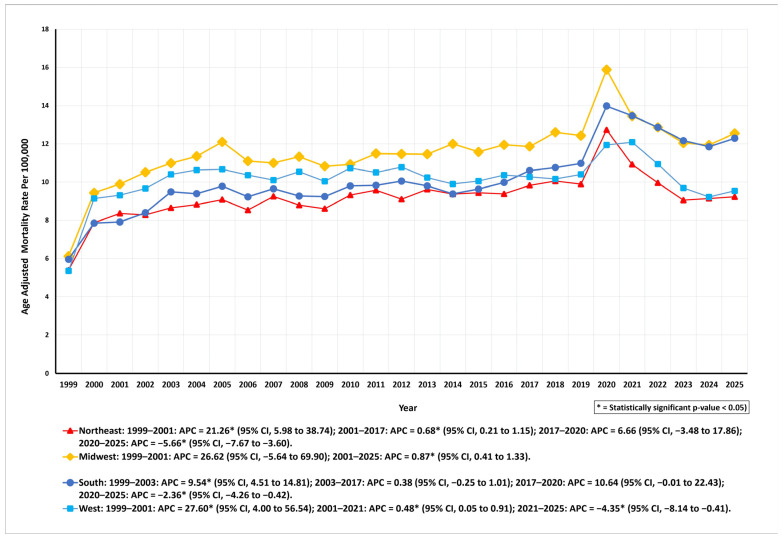
Census region-stratified age-adjusted mortality rates per 100,000 for Co-Recorded Parkinson’s disease- and Dementia-related mortality in United States adults aged ≥45 years, 1999–2025.

In the West region, AAMRs increased from 5.35 (95% CI: 5.01–5.68) in 1999 to 9.54 (95% CI: 9.22–9.88) in 2025 (AAPC: 1.57; 95% CI: −0.05–3.22, *p* = 0.058). AAMR rose significantly from 1999 to 2001 (APC: 27.60; 95% CI: 4.00–56.54, *p* = 0.022) and then increased steadily from 2001 to 2021 (APC: 0.48; 95% CI: 0.05–0.91, *p* = 0.030). From 2021 to 2025, mortality rates declined significantly (APC: −4.35; 95% CI: −8.14 to −0.41, *p* = 0.033) (Figure 5) (Supplemental Tables S2 and S7).

### 3.6. Gender-Stratified AAMR Trends for Parkinson’s and Dementia

In females, AAMRs increased from 4.31 (95% CI: 4.15–4.48) in 1999 to 7.34 (95% CI: 7.16–7.52) in 2025 (AAPC: 1.68; 95% CI: 0.32–3.05, *p* = 0.015). AAMR increased significantly from 1999 to 2001 (APC: 25.09; 95% CI: 11.17–40.75, *p* = 0.001), followed by a non-significant change from 2001 to 2020. Rates then declined significantly from 2020 to 2025 (APC: −4.52; 95% CI: −6.31 to -2.69, *p* = 0.0001).

In males, AAMRs increased from 8.27 (95% CI: 7.96–8.58) in 1999 to 16.37 (95% CI: 16.06–16.69) in 2025 (AAPC: 2.14; 95% CI: 0.66–3.65, *p* = 0.005). AAMR increased significantly from 1999 to 2001 (APC: 24.02; 95% CI: 7.32–43.31, *p* = 0.006), and continued to increase from 2001 to 2017 (APC: 0.47; 95% CI: 0.02–0.93, *p* = 0.042). From 2017 to 2020, AAMR showed a non-significant increase, and then declined significantly from 2020 to 2025 (APC: −3.38; 95% CI: −5.13 to −1.60, *p* = 0.001) (Figure 1) (Supplemental Tables S1 and S3).

### 3.7. State-Stratified AAMR Trends for Parkinson’s and Dementia

From 1999 to 2020, the state with the highest average AAMR was Minnesota, with an AAMR of 15.50, while the state with the lowest average AAMR was Nevada, with an AAMR of 6.58. The states in the top 90th percentile included Minnesota, Vermont, Iowa, and Nebraska, while the states in the lowest 10th percentile included Nevada, the District of Columbia, Alaska, and Wyoming.

From 2021 to 2025, the state with the highest average AAMR was Kentucky (20.42), while the state with the lowest average AAMR was Hawaii (5.73). States falling within the top percentile range included Nebraska, Oregon, South Carolina, Oklahoma, and Colorado, all reflecting consistently elevated AAMRs. In contrast, the lowest average AAMR was observed in Hawaii, followed by the District of Columbia, New Jersey, Alabama, and Arizona (Supplementary Figure S1) (Supplemental Table S8).

### 3.8. Place of Death-Stratified AAMR Trends for Parkinson’s and Dementia

Place-of-death analysis revealed that the majority of deaths occurred in nursing homes or long-term care facilities (50.9%), followed by home (23.3%) and hospital inpatient settings (12.5%). Hospice facilities accounted for 5.4% of deaths, while other locations contributed smaller proportions (Supplementary Figure S2) (Supplemental Table S9).

#### 3.8.1. Overall Trend Excluding 2025 Provisional Data

Overall, AAMRs increased from 5.75 (95% CI: 5.60–5.90) in 1999 to 10.75 (95% CI: 10.59–10.92) in 2024 (AAPC: 2.18; 95% CI: 1.05–3.32, *p* = 0.000147). AAMR increased sharply from 1999 to 2001 (APC: 24.28; 95% CI: 12.29–37.54, *p* = 0.0004), followed by a slower but significant rise from 2001 to 2017 (APC: 0.45; 95% CI: 0.12–0.78, *p* = 0.011). Rates further increased from 2017 to 2020 (APC: 8.11; 95% CI: 1.05–15.66, *p* = 0.026). From 2020 to 2024, mortality rates declined notably (APC: −4.93; 95% CI: −6.80 to −3.01, *p* = 0.0001 (Supplementary Tables S1 and S10).

#### 3.8.2. COVID-19

AAMRs increased from 5.75 (95% CI: 5.60–5.90) in 1999 to 10.98 (95% CI: 10.80–11.15) in 2019 (AAPC: 2.74; 95% CI: 1.70–3.79, *p* < 0.000001). AAMR increased sharply from 1999 to 2001 (APC: 23.19; 95% CI: 10.67–37.12, *p* = 0.0008) and then continued to rise more gradually from 2001 to 2019 (APC: 0.69; 95% CI: 0.43–0.95, *p* = 0.0004 (Supplementary Tables S1 and S11).

#### 3.8.3. Sensitivity Analysis

For UCD dementia, AAMRs increased from 78.70 (95% CI: 78.13–79.26) in 1999 to 209.38 (95% CI: 208.64–210.12) in 2025 (AAPC: 3.67; 95% CI: 3.30–4.05, *p* < 0.000001). Mortality rates increased significantly from 1999 to 2010 (APC: 7.14; 95% CI: 6.31–7.98, *p* < 0.000001), followed by a slower but continued significant increase from 2010 to 2025 (APC: 1.20; 95% CI: 0.86–1.54, *p* < 0.000001).

For UCD Parkinson’s disease (PD), AAMRs increased from 15.28 (95% CI: 15.04–15.53) in 1999 to 28.43 (95% CI: 28.17–28.71) in 2025 (AAPC: 2.09; 95% CI: 1.67–2.52, *p* < 0.000001). Mortality rates increased significantly from 1999 to 2014 (APC: 1.74; 95% CI: 1.40–2.08, *p* < 0.000001) and rose more rapidly from 2014 to 2020 (APC: 4.60; 95% CI: 3.14–6.08, *p* = 0.000002). From 2020 to 2025, mortality rates remained stable (Supplementary Figure S3) (Supplemental Tables S1 and S12).

## 4. Discussion

**Figure 6:**
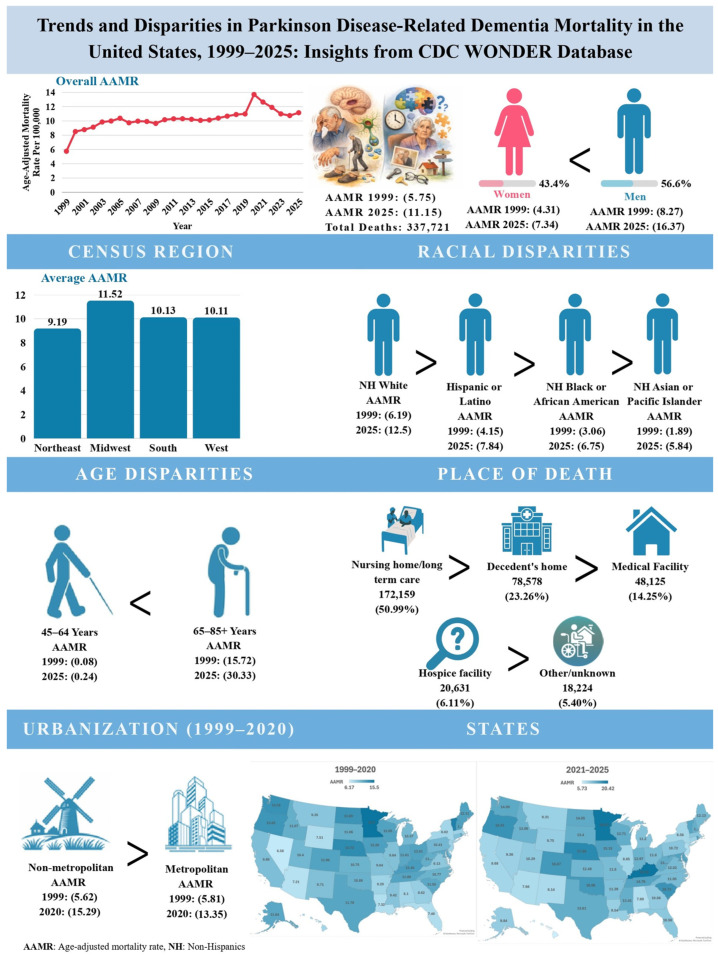
Central Illustration.

This comprehensive nationwide analysis identified changing mortality patterns involving co-recorded Parkinson’s disease (PD) and dementia across the United States from 1999 to 2025. Mortality rates increased rapidly during the early study period, followed by a substantially slower rate of increase through 2017, a transient spike during the COVID-19 pandemic period, and a subsequent decline in mortality rates was observed after 2020 (Figure 6). These findings highlight the growing burden of overlapping neurodegenerative disorders and underscore the importance of evaluating multiple-cause mortality, which better reflects the clinical intricacy of comorbid states compared with single underlying-cause approaches [13,14].

The observed temporal pattern in mortality likely reflects a combination of demographic, clinical, and reporting-related factors. Aging populations stand as the primary factor, given the exponential rise in Parkinson’s disease and dementia occurrence with advancing years [1,15]. Meanwhile, progress in PD treatment has prolonged survival, raising the chances of patients reaching advanced phases marked by cognitive decline and dementia [16]. Longitudinal cohort studies have demonstrated that up to 80% of patients with PD eventually develop dementia, which is strongly associated with increased mortality [4,17]. Enhanced detection and recording of dementia in PD cases may also play a role in these rising trends. Historical data suggest dementia was often missed in PD patients, especially in earlier periods, and greater awareness could partly account for the temporal increase [17]. Notably, Parkinson’s disease dementia presents as a more severe manifestation, tied to elevated hospitalization, institutionalization, and death rates relative to Parkinson’s disease without cognitive deficits [18].

The observed increase in mortality during the early study period followed by a substantially slower rate of increase in later years may reflect several factors beyond disease burden alone. Potential contributors include increased diagnostic awareness of Parkinson’s disease and dementia, evolving death-certification and coding practices, improved recognition of neurodegenerative disorders, and demographic aging of the population. Administrative and reporting changes over time may also have influenced national mortality trends derived from death-certificate data [19,20]. These considerations suggest that the observed temporal patterns should not be interpreted solely as evidence of a continuously increasing disease burden.

The marked increase in mortality observed during 2020 likely reflects multiple pandemic-related factors. Older adults with neurodegenerative disorders experienced disproportionately high COVID-19 mortality, particularly among nursing-home residents and individuals with functional dependence. Disruptions in outpatient neurologic care, delayed access to medical services, social isolation, and changes in death-certificate reporting practices during the pandemic may also have contributed to transient increases in mortality coding involving Parkinson’s disease and dementia [21,22]. The subsequent decline after 2020 should therefore be interpreted cautiously and may partly reflect normalization of healthcare systems and provisional reporting for recent years.

Predictably, mortality peaked among older adults, showcasing the accumulating toll of neurodegeneration. Yet, the uptick in fatalities among those aged 45–64 merits attention, possibly signaling shifting disease dynamics. Earlier-onset PD has been linked to prolonged illness duration and a heightened cumulative dementia risk [23]. Moreover, better recognition of cognitive issues in younger PD cohorts could be amplifying this pattern. Recent studies propose that mild cognitive impairment in PD strongly forecasts progression to dementia and mortality, underlining the necessity of early identification and tracking [24]. These insights advocate for regular cognitive assessments in PD patients of all ages.

The elevated mortality in males aligns with prior epidemiological findings showing both higher PD incidence and poorer outcomes in men [3,25]. Biological factors, like sex-specific hormonal effects and divergent neuroinflammatory reactions, might heighten male susceptibility [26]. Men may also carry heavier comorbidity burdens and exhibit distinct healthcare-seeking behaviors that worsen results.

Pronounced racial and ethnic disparities were observed, with higher mortality among non-Hispanic White individuals. This pattern has been described in prior studies and may reflect differences in longevity, diagnostic practices, and healthcare access [27]. White populations may have greater access to neurologic evaluation, leading to higher detection and documentation of dementia in PD. Conversely, lower documented mortality in minority groups could signal underdiagnosis and care-access inequities. Research indicates Black and Hispanic individuals are less likely to obtain specialist PD and dementia care, potentially leading to missed diagnoses and underreporting [28]. Structural inequalities, including socioeconomic hurdles and healthcare barriers, likely play pivotal roles in these gaps.

Geographic and urban–rural variations in this study probably mirror disparities in healthcare access and population characteristics. Rural residents often face limited neurologist availability and specialized services, resulting in delayed diagnoses and subpar management [29]. Past studies confirm that neurologist access correlates with better outcomes and reduced PD mortality [30]. Regional mortality fluctuations further expose stark divides in the burden of co-recorded Parkinson’s disease and dementia. Our analysis identified the Midwest and South with the highest AAMRs, while the Northeast and West posted relatively lower figures. These contrasts likely arise from differences in aging demographics, comorbidity burdens, and specialized neurology care access. Areas with sizable rural populations, notably in the Midwest and South, may endure delayed diagnoses and scarce movement disorder specialists, exacerbating outcomes [29,30]. In comparison, regions with denser healthcare networks, like the Northeast, may gain from earlier detection and multidisciplinary care. State-level diversity further underscores the impact of local factors, such as healthcare systems and socioeconomic contexts. Together, these findings highlight the urgent need for tailored, region-specific approaches to enhance care access and mitigate geographic imbalances in neurodegenerative disease results.

Regional discrepancies in environmental exposures, lifestyle elements, and healthcare frameworks may also feed into mortality rate variability. The predominance of deaths in nursing homes and long-term care facilities reflects the advanced stage of PD-related dementia. Patients with PD dementia often necessitate institutionalization due to profound cognitive and functional decline [31]. This reality calls for expanded long-term care resources and stronger caregiver support networks. Early palliative care integration has proven to elevate life quality and may reshape end-of-life care trajectories in neurodegenerative illnesses [32].

The co-occurrence of PD and dementia points to intertwined neurodegenerative mechanisms, involving α-synuclein pathology and coexisting Alzheimer-type changes [33]. This dual pathology correlates with accelerated disease advancement and poorer clinical consequences. Neuroinflammation, synaptic disruption, and extensive cortical involvement likely exacerbate mortality risk in these patients [34].

This study benefits from a large, nationally representative dataset and the use of multiple-cause mortality data, which provides a more comprehensive assessment of disease burden. Nonetheless, death certificate records are prone to misclassification, and evolving coding conventions may skew observed patterns [35]. The absence of clinical granularity also restricts evaluations of disease severity and treatment impacts.

## 5. Limitations

This study has several limitations. Death-certificate data are susceptible to misclassification bias and temporal variation in diagnostic and coding practices. Because this was a population-level multiple-cause mortality analysis, the study could not estimate individual-level mortality risk or adjust for potential confounders such as comorbidities, socioeconomic status, healthcare access, or treatment differences. In addition, death-certificate data cannot determine disease onset, diagnostic timing, disease severity, causal relationships, or whether dementia developed before or after Parkinson’s disease. The dementia ICD-10 codes included heterogeneous neurodegenerative and vascular conditions that may not represent clinically confirmed Parkinson’s disease dementia. Mortality trends during 2020–2021 may also have been influenced by the COVID-19 pandemic, including excess mortality, healthcare disruption, and changes in death-certification practices. Furthermore, recent mortality data may be subject to reporting delays or provisional classification, which could influence interpretation of post-pandemic temporal trends. Finally, although age-adjusted mortality rates standardized to the 2000 U.S. population were used, demographic and population changes over time may still influence long-term mortality patterns.

## 6. Strengths

Despite these limitations, multiple-cause mortality analyses provide valuable surveillance insight into the evolving public-health burden of neurodegenerative diseases. Evaluating conditions recorded anywhere on death certificates may better capture the complexity of advanced neurodegenerative illness than underlying-cause analyses alone.

## 7. Conclusions

Mortality involving co-recorded Parkinson’s disease and dementia-related diagnoses recorded on death certificates demonstrated changing temporal patterns in the United States, characterized by an early increase, a prolonged period of slower growth, and a transient rise during the COVID-19 pandemic. These findings represent population-level mortality burden involving co-recorded conditions rather than mortality risk among individuals diagnosed with Parkinson’s disease or dementia. Future studies using patient-level clinical data are needed to better characterize the epidemiology and clinical implications of co-recorded Parkinson’s disease and dementia mortality.

## Data Availability

The dataset analyzed in this study is publicly available on the CDC WONDER online database (https://wonder.cdc.gov/, accessed on 24 March 2026). No special access permissions were required.

## Supplementary Materials

The following supporting information can be downloaded at https://www.mdpi.com/article/10.3390/neurosci7030066/s1: Table S1: Annual percent change (APC) and Average annual percent change (AAPC) of co-recorded Parkinson disease and dementia related mortality stratified by overall, sex, COVID, overall excluding 2025 provisional data, sensitivity analysis among adults aged ≥45 in the United States, 1999–2025; Table S2: Annual percent change (APC) and Average annual percent change (AAPC) of co-recorded Parkinson disease and dementia related mortality stratified by Race, age group, urbanization, census region among adults aged ≥45 in the United States, 1999–2025; Table S3: National Trends and Demographic Disparities in Mortality Involving Co-Recorded Parkinson Disease and Dementia in the United States stratified by Overall and Sex, 1999–2025; Table S4: National Trends and Demographic Disparities in Mortality Involving Co-Recorded Parkinson Disease and Dementia in the United States stratified by Age group, 1999–2025; Table S5: National Trends and Demographic Disparities in Mortality Involving Co-Recorded Parkinson Disease and Dementia in the United States stratified by Race 1999–2025; Table S6: National Trends and Demographic Disparities in Mortality Involving Co-Recorded Parkinson Disease and Dementia in the United States stratified by Urbanization, 1999–2020; Table S7: National Trends and Demographic Disparities in Mortality Involving Co-Recorded Parkinson Disease and Dementia in the United States stratified by Census region, 1999–2025; Table S8: National Trends and Demographic Disparities in Mortality Involving Co-Recorded Parkinson Disease and Dementia in the United States stratified by State, 1999–2025; Table S9: Percentage distribution of place of death in the United States (1999–2025), with Involving Co-Recorded Parkinson Disease and Dementia related mortality highlighted; Table S10: National Trends and Demographic Disparities in Mortality Involving Co-Recorded Parkinson Disease and Dementia in the United States stratified by overall excluding 2025 provisional data, 1999–2024; Table S11: National Trends and Demographic Disparities in Mortality Involving Co-Recorded Parkinson Disease and Dementia in the United States stratified by Before COVID, 1999–2019; Table S12: National Trends and Demographic Disparities in Mortality Involving Co-Recorded Parkinson Disease and Dementia in the United States stratified by sensitivity analysis, 1999–2025; Figure S1. National Trends and Demographic Disparities in Mortality Involving Co-Recorded Parkinson Disease and Dementia in the United States stratified by States adults aged ≥45 years, 1999–2025; Figure S2. Place-of-death trends in the U.S. (1999–2025), highlighting mortality Dementia Involving Co-Recorded Parkinson Disease and Dementia; Figure S3. Sensitivity analysis stratified age-adjusted mortality rates per 100,000 for Co-Recorded Parkinson Disease and Dementia related mortality in United States adults aged ≥45 years, 1999–2025.

## Abbreviations

AAMR	Age-Adjusted Mortality Rate
APC	Annual Percent Change
AAPC	Average Annual Percent Change
CDC	Centers for Disease Control and Prevention
ICD-10	International Classification of Diseases, 10th Revision
NH	Non-Hispanic

## References

[1] GBD 2019 Dementia Forecasting Collaborators (2022). Estimation of the global prevalence of dementia in 2019 and forecasted prevalence in 2050: An analysis for the Global Burden of Disease Study 2019. Lancet Public Health.

[2] Dorsey E.R., Sherer T., Okun M.S., Bloem B.R. (2018). The emerging evidence of the Parkinson pandemic. J. Park. Dis..

[3] Hely M.A., Reid W.G.J., Adena M.A., Halliday G.M., Morris J.G.L. (2008). The Sydney multicenter study of Parkinson’s disease: The inevitability of dementia at 20 years. Mov. Disord..

[4] Feigin V.L., Vos T., Alahdab F., Amit A.M.L., Bärnighausen T.W., Beghi E., Beheshti M., Chavan P.P., Criqui M.H., GBD 2017 US Neurological Disorders Collaborators (2021). Burden of neurological disorders across the US from 1990–2017: A Global Burden of Disease Study. JAMA Neurol..

[5] Aarsland D., Batzu L., Halliday G.M., Geurtsen G.J., Ballard C., Chaudhuri K.R., Weintraub D. (2021). Parkinson disease-associated cognitive impairment. Nat. Rev. Dis. Primers.

[6] Kalia L.V., Lang A.E. (2015). Parkinson’s disease. Lancet.

[7] Matthews K.A., Xu W., Gaglioti A.H., Holt J.B., Croft J.B., Mack D., McGuire L.C. (2019). Racial and ethnic estimates of Alzheimer’s disease and related dementias in the United States (2015–2060) in adults aged ≥65 years. Alzheimers Dement..

[8] Centers for Disease Control and Prevention (CDC) Wide-Ranging Online Data for Epidemiologic Research (CDC WONDER): Multiple Cause of Death Files, 1999–2025.

[9] Alam T., Burney W., Kamal S., Kamal A., Abufatima I.O., Ali U., Mukhlis M., Khatri A., Usman N., Aqeel N. (2026). Long-term trends in Parkinson’s disease and associated mental health disorders: Insights from the CDC WONDER database, 1999–2023. Brain Behav..

[10] Bhattacharyya J., Barnes L.L., Chen Y., Gianattasio K.Z., Grodstein F., James B.D., Marquez D.X., Moghtaderi A., Prather C., Rein D.B. (2025). Evaluating linked ICD-10 Medicare claims data as a method of dementia case ascertainment in research settings. Alzheimers Dement..

[11] von Elm E., Altman D.G., Egger M., Pocock S.J., Gøtzsche P.C., Vandenbroucke J.P. (2007). The STROBE statement: Guidelines for reporting observational studies. PLoS Med..

[12] National Cancer Institute Joinpoint Regression Program.

[13] Redelings M.D., Wise M., Sorvillo F. (2007). Using multiple cause-of-death data to investigate associations and causality between conditions listed on the death certificate. Am. J. Epidemiol..

[14] Ntonghanwah F., Setlhare K., Amey A.K.A. (2014). Modeling severity of tuberculosis as a multiple cause of death in South Africa. J. Tuberc. Res..

[15] de Lau L.M.L., Breteler M.M.B. (2006). Epidemiology of Parkinson’s disease. Lancet Neurol..

[16] Aarsland D., Kurz M.W. (2010). The epidemiology of dementia associated with Parkinson’s disease. Brain Pathol..

[17] Gallagher J., Gochanour C., Caspell-Garcia C., Dobkin R.D., Aarsland D., Alcalay R.N., Barrett M.J., Chahine L., Chen-Plotkin A.S., Coffey C.S. (2024). Long-term dementia risk in Parkinson disease. Neurology.

[18] Goetz C.G., Emre M., Dubois B. (2008). Parkinson’s disease dementia: Definitions, guidelines, and research perspectives in diagnosis. Ann. Neurol..

[19] Lampropoulos I.C., Malli F., Sinani O., Gourgoulianis K.I., Xiromerisiou G. (2022). Worldwide trends in mortality related to Parkinson’s disease in the period of 1994–2019: Analysis of vital data from the WHO Mortality Database. Front. Neurol..

[20] Gao L., Calloway R., Zhao E., Brayne C., Matthews F.E., Medical Research Council Cognitive Function and Ageing Collaboration (2018). Accuracy of death certification of dementia in population-based samples of older people: Analysis over time. Age Ageing.

[21] Williamson E.J., Walker A.J., Bhaskaran K., Bacon S., Bates C., Morton C.E., Curtis H.J., Mehrkar A., Evans D., Inglesby P. (2020). Factors associated with COVID-19-related death using OpenSAFELY. Nature.

[22] Reyes-Bueno J., Mena-Vázquez N., Ojea-Ortega T., Gonzalez-Sotomayor M., Cabezudo-Garcia P., Ciano-Petersen N., Pons-Pons G., Castro-Sánchez M., Serrano-Castro P. (2020). Case fatality of COVID-19 in patients with neurodegenerative dementia. Neurologia.

[23] Chang T.Y., Yang C.P., Chen Y.H., Lin C.H., Chang M.H. (2021). Age-stratified risk of dementia in Parkinson’s disease: A nationwide population-based retrospective cohort study in Taiwan. Front. Neurol..

[24] Aarsland D., Creese B., Politis M., Chaudhuri K.R., Ffytche D.H., Weintraub D., Ballard C. (2017). Cognitive decline in Parkinson disease. Nat. Rev. Neurol..

[25] Cerri S., Mus L., Blandini F. (2019). Parkinson’s disease in women and men: What’s the difference?. J. Park. Dis..

[26] Müller L., Di Benedetto S., Müller V. (2025). Influence of biological sex on neuroinflammatory dynamics in the aging brain. Front. Aging Neurosci..

[27] Aamodt W.W., Willis A.W., Dahodwala N. (2023). Racial and ethnic disparities in Parkinson disease: A call to action. Neurol. Clin. Pract..

[28] University of California, Davis Health (2024). Study Finds Disparities in Diagnosis and Treatment of Dementia. https://health.ucdavis.edu/news/headlines/study-finds-disparities-in-diagnosis-and-treatment-of-dementia/2024/01.

[29] American Academy of Neurology Press Release: Dementia and Parkinson’s Disease Findings. https://www.aan.com/PressRoom/Home/PressRelease/3843.

[30] Willis A.W., Schootman M., Tran R., Kung N., Evanoff B.A., Perlmutter J.S., Racette B.A. (2012). Neurologist-associated reduction in PD-related hospitalizations and health care expenditures. Neurology.

[31] Pigott J.S., Bloem B.R., Lorenzl S., Meissner W.G., Odin P., Ferreira J.J., Dodel R., Schrag A. (2024). The care needs of patients with cognitive impairment in late-stage Parkinson’s disease. J. Geriatr. Psychiatry Neurol..

[32] Walter H.A.W., Seeber A.A., Willems D.L., de Visser M. (2019). The role of palliative care in chronic progressive neurological diseases. Front. Neurol..

[33] Irwin D.J., Lee V.M., Trojanowski J.Q. (2013). Parkinson’s disease dementia: Convergence of α-synuclein, tau and amyloid-β pathologies. Nat. Rev. Neurosci..

[34] van Wetering J., Geut H., Bol J.J., Galis Y., Timmermans E., Twisk J.W., Hepp D.H., Morella M.L., Pihlstrom L., Lemstra A.W. (2024). Neuroinflammation is associated with Alzheimer’s disease co-pathology in dementia with Lewy bodies. Acta Neuropathol. Commun..

[35] Bishop K., Balogun S., Eynstone-Hinkins J., Moran L., Martin M., Banks E., Rao C., Joshy G. (2023). Analysis of multiple causes of death: A review of methods and practices. Epidemiology.

