# Supplementary material for: National Trends and Demographic Disparities in Mortality Involving Co-Recorded Parkinson’s Disease and Dementia in the United States, 1999–2025: A CDC WONDER Analysis
Source: NeuroSci. 2026 Jun 10;7(3):66. doi: 10.3390/neurosci7030066 (PMC13304537; doi:10.3390/neurosci7030066)
Supplement: Supplementary file 1 [file neurosci-07-00066-s001.zip › neurosci-4270103-supplementary.pdf]

NeuroSci

MDPI

Table S1. Annual Percent Change (APC) and Average Annual Percent Change (AAPC) of Co-Recorded Parkinson's Disease and Dementia-Related Mortality Stratified by Overall Population, Sex, COVID-19 Period, Overall Population Excluding 2025 Provisional Data, and Sensitivity Analysis among Adults Aged ≥45 Years in the United States, 1999–2025.

Table S2. Annual Percent Change (APC) and Average Annual Percent Change (AAPC) of Co-Recorded Parkinson's Disease and Dementia-Related Mortality Stratified by Race, Age Group, Urbanization, and Census Region among Adults Aged ≥45 Years in the United States, 1999–2025.

Table S3. National Trends and Demographic Disparities in Mortality Involving Co-Recorded Parkinson's Disease and Dementia in the United States, Stratified by Overall Population and Sex, 1999–2025.

Table S4. National Trends and Demographic Disparities in Mortality Involving Co-Recorded Parkinson's Disease and Dementia in the United States, Stratified by Age Group, 1999–2025.

Table S5. National Trends and Demographic Disparities in Mortality Involving Co-Recorded Parkinson's Disease and Dementia in the United States, Stratified by Race, 1999–2025.

Table S6. National Trends and Demographic Disparities in Mortality Involving Co-Recorded Parkinson's Disease and Dementia in the United States, Stratified by Urbanization, 1999–2020.

Table S7. National Trends and Demographic Disparities in Mortality Involving Co-Recorded Parkinson's Disease and Dementia in the United States, Stratified by Census Region, 1999–2025.

Table S8. National Trends and Demographic Disparities in Mortality Involving Co-Recorded Parkinson's Disease and Dementia in the United States, Stratified by State, 1999–2025.

Table S9. Percentage Distribution of Place of Death in the United States (1999–2025), Highlighting Mortality Involving Co-Recorded Parkinson's Disease and Dementia.

Table S10. National Trends and Demographic Disparities in Mortality Involving Co-Recorded Parkinson's Disease and Dementia in the United States, Stratified by Overall Population Excluding 2025 Provisional Data, 1999–2024.

Table S11. National Trends and Demographic Disparities in Mortality Involving Co-Recorded Parkinson's Disease and Dementia in the United States, Stratified by Pre-COVID-19 Period, 1999–2019.

Table S12. National Trends and Demographic Disparities in Mortality Involving Co-Recorded Parkinson's Disease and Dementia in the United States, Stratified by Sensitivity Analysis, 1999–2025.

Figure S1. National Trends and Demographic Disparities in Mortality Involving Co-Recorded Parkinson's Disease and Dementia among Adults Aged ≥45 Years in the United States, Stratified by State, 1999–2025.

Figure S2. Place-of-death trends in the United States (1999–2025), highlighting mortality involving co-recorded Parkinson's Disease and dementia.

Figure S3. Sensitivity analysis of age-adjusted mortality rates per 100,000 for co-recorded Parkinson's Disease and dementia-related mortality among adults aged ≥45 years in the United States, 1999–2025.

https://doi.org/10.3390/xxxxx

Table S1: Annual percent change (APC) and Average annual percent change (AAPC) of co-recorded Parkinson's disease and Dementia-related mortality stratified by overall, sex, COVID, overall excluding 2025 provisional data, sensitivity analysis among adults aged ≥45 in the United States, 1999–2025.

Year	APC (95% CI)	p-value for APC	AAPC (95% CI)	p-value for AAPC
		**Overall**		
**1999-2001**	24.23 (9.60 to 40.80)	0.002065	2.07 (0.73 to 3.42)	0.002343
**2001-2017**	0.46 (0.05 to 0.87)	0.029809		
**2017-2020**	7.60 (-1.01 to 16.96)	0.081106		
**2020-2025**	-3.82 (-5.52 to -2.09)	0.000284		
		**Sex**		
		**Women**		
**1999-2001**	24.02 (7.32 to 43.31)	0.006114	2.14 (0.66 to 3.65)	0.004462
**2001-2017**	0.47 (0.02 to 0.93)	0.041764		
**2017-2020**	7.52 (-1.67 to 17.58)	0.104814		
**2020-2025**	-3.38 (-5.13 to -1.60)	0.001063		
		**Men**		
**1999-2001**	25.09 (11.17 to 40.75)	0.000985	1.68 (0.32 to 3.05)	0.015211
**2001-2017**	0.04 (-0.37 to 0.46)	0.821846		
**2017-2020**	7.22 (-2.06 to 17.38)	0.122268		
**2020-2025**	-4.52 (-6.31 to -2.69)	0.000095		
		**Overall excluding 2025 provisional data**		
**1999-2001**	24.28 (12.29 to 37.54)	0.000369	2.18 (1.05 to 3.32)	0.000147
**2001-2017**	0.45 (0.12 to 0.78)	0.011346		
**2017-2020**	8.11 (1.05 to 15.66)	0.026427		
**2020-2024**	-4.93 (-6.80 to -3.01)	0.000074		
		Before **COVID**		
**1999-2001**	23.19 (10.67 to 37.12)	0.000793	2.74 (1.70 to 3.79)	< 0.000001
**2001-2019**	0.69 (0.43 to 0.95)	0.000039		
		**Sensitivity analysis**		
		**UCD Dementia**		
**1999-2010**	7.14 (6.31 to 7.98)	< 0.000001	3.67 (3.30 to 4.05)	< 0.000001
**2010-2025**	1.20 (0.86 to 1.54)	< 0.000001		
		**UCD PD**		
**1999-2014**	1.74 (1.40 to 2.08)	< 0.000001	2.09 (1.67 to 2.52)	< 0.000001
**2014-2020**	4.60 (3.14 to 6.08)	0.000002		
**2020-2025**	0.20 (-1.04 to 1.46)	0.738401		

Table S2: Annual percent change (APC) and Average annual percent change (AAPC) of co-recorded Parkinson's disease and Dementia-related mortality stratified by Race, age group, urbanization, census region among adults aged ≥45 in the United States, 1999–2025.

Year	APC (95% CI)	p-value for APC	AAPC (95% CI)	p-value for AAPC
		**Race**		
		**NH Asian or Pacific Islander**		
**1999-2002**	26.86 (-11.28 to 81.39)	0.181533	3.17 (-0.82 to 7.32)	0.120927
**2002-2025**	0.43 (-0.28 to 1.14)	0.225735		
		**NH Black or African American**		
**1999-2002**	16.79 (-4.28 to 42.49)	0.119842	2.90 (0.63 to 5.21)	0.011890
**2002-2025**	1.21 (0.61 to 1.81)	0.000345		
		**NH White**		
**1999-2001**	23.90 (8.98 to 40.87)	0.002718	2.29 (0.90 to 3.70)	0.001162
**2001-2017**	0.63 (0.21 to 1.05)	0.005422		
**2017-2020**	7.85 (-1.24 to 17.78)	0.087604		
**2020-2025**	-3.28 (-4.99 to -1.55)	0.001086		
		**Hispanic or Latino**		
**1999-2006**	6.78 (4.16 to 9.47)	0.000040	1.74 (0.43 to 3.08)	0.009329
**2006-2017**	0.29 (-0.66 to 1.24)	0.533385		
**2017-2020**	8.45 (-1.51 to 19.41)	0.093100		
**2020-2025**	-5.53 (-7.38 to -3.64)	0.000016		
		**Age group**		
		**45-64 Years**		
**1999-2018**	4.37 (3.13 to 5.63)	< 0.000001	3.55 (0.33 to 6.88)	0.030681
**2018-2021**	11.92 (-13.85 to 45.39)	0.379004		
**2021-2025**	-5.90 (-13.17 to 1.97)	0.129372		
		**65-74 Years**		
**1999-2001**	22.76 (1.47 to 48.52)	0.036464	2.65 (1.01 to 4.31)	0.001430
**2001-2015**	0.21 (-0.58 to 1.00)	0.587548		
**2015-2020**	7.72 (3.80 to 11.80)	0.000611		
**2020-2025**	-2.58 (-4.77 to -0.34)	0.026607		
		**75-84 Years**		
**1999-2001**	23.56 (6.93 to 42.77)	0.006845	2.16 (0.58 to 3.76)	0.007317
**2001-2017**	0.56 (0.07 to 1.05)	0.028775		
**2017-2020**	7.51 (-2.83 to 18.96)	0.148521		
**2020-2025**	-3.43 (-5.39 to -1.42)	0.002399		
		**85+ Years**		
**1999-2002**	15.73 (5.15 to 27.38)	0.005249	1.64 (-0.15 to 3.47)	0.073120
**2002-2017**	0.11 (-0.55 to 0.77)	0.740170		
**2017-2020**	6.43 (-6.01 to 20.53)	0.303508		
**2020-2025**	-4.25 (-6.90 to -1.52)	0.004716		
		**Urbanization**		
		**Metropolitan**		
**2001-2018**	0.44 (0.13 to 0.76)	0.009498	3.54 (2.35 to 4.74)	< 0.000001
**2018-2020**	11.95 (4.54 to 19.90)	0.003314		
		**Non-metropolitan**		
**1999-2001**	26.05 (6.44 to 49.28)	0.010847	4.23 (2.54 to 5.96)	0.000001
**2001-2016**	0.84 (0.20 to 1.48)	0.013378		
**2016-2020**	7.32 (3.70 to 11.07)	0.000594		
		**Census Region**		
		**Northeast**		
**1999-2001**	21.26 (5.98 to 38.74)	0.007890	1.53 (0.01 to 3.07)	0.047849
**2001-2017**	0.68 (0.21 to 1.15)	0.007488		
**2017-2020**	6.66 (-3.48 to 17.86)	0.190159		
**2020-2025**	-5.66 (-7.67 to -3.60)	0.000031		

		**Midwest**		
**1999-2001**	26.62 (-5.64 to 69.90)	0.110225	2.65 (0.44 to 4.90)	0.018510
**2001-2025**	0.87 (0.41 to 1.33)	0.000681		
		**South**		
**1999-2003**	9.54 (4.51 to 14.81)	0.000814	2.34 (0.96 to 3.73)	0.000811
**2003-2017**	0.38 (-0.25 to 1.01)	0.219677		
**2017-2020**	10.64 (-0.01 to 22.43)	0.050280		
**2020-2025**	-2.36 (-4.26 to -0.42)	0.020429		
		**West**		
**1999-2001**	27.60 (4.00 to 56.54)	0.021974	1.57 (-0.05 to 3.22)	0.058208
**2001-2021**	0.48 (0.05 to 0.91)	0.029500		
**2021-2025**	-4.35 (-8.14 to -0.41)	0.032736		

NeuroSci

Table S3. National Trends and Demographic Disparities in Mortality Involving Co-Recorded Parkinson's Disease and Dementia in the United States stratified by Overall and Sex, 1999–2025.

Year	Overall		Women		Men	
	Deaths	AAMR (95% CI)	Deaths	AAMR (95% CI)	Deaths	AAMR (95% CI)
**1999**	5452	5.75 (5.60 to 5.90)	2641	4.31 (4.15 to 4.48)	2811	8.27 (7.96 to 8.58)
**2000**	8158	8.51 (8.33 to 8.70)	3976	6.42 (6.22 to 6.62)	4182	12.22 (11.84 to 12.60)
**2001**	8551	8.78 (8.60 to 8.97)	4077	6.53 (6.33 to 6.73)	4474	12.70 (12.32 to 13.07)
**2002**	9016	9.13 (8.94 to 9.32)	4314	6.82 (6.62 to 7.03)	4702	13.23 (12.85 to 13.62)
**2003**	9863	9.85 (9.65 to 10.04)	4752	7.42 (7.20 to 7.63)	5111	13.98 (13.60 to 14.37)
**2004**	10136	9.99 (9.79 to 10.18)	4752	7.34 (7.13 to 7.55)	5384	14.42 (14.03 to 14.81)
**2005**	10727	10.38 (10.18 to 10.57)	5107	7.78 (7.56 to 7.99)	5620	14.63 (14.24 to 15.01)
**2006**	10263	9.74 (9.55 to 9.93)	4799	7.22 (7.02 to 7.43)	5464	13.92 (13.55 to 14.29)
**2007**	10689	9.97 (9.78 to 10.16)	5040	7.51 (7.31 to 7.72)	5649	14.05 (13.68 to 14.41)
**2008**	10801	9.92 (9.73 to 10.11)	4928	7.22 (7.01 to 7.42)	5873	14.24 (13.87 to 14.60)
**2009**	10695	9.65 (9.47 to 9.84)	4930	7.15 (6.95 to 7.36)	5765	13.70 (13.35 to 14.06)
**2010**	11434	10.17 (9.99 to 10.36)	5172	7.35 (7.15 to 7.55)	6262	14.60 (14.23 to 14.96)
**2011**	11910	10.30 (10.11 to 10.48)	5462	7.62 (7.41 to 7.82)	6448	14.45 (14.10 to 14.81)
**2012**	12218	10.31 (10.13 to 10.50)	5454	7.44 (7.24 to 7.64)	6764	14.80 (14.45 to 15.16)
**2013**	12377	10.23 (10.04 to 10.41)	5368	7.21 (7.01 to 7.40)	7009	14.77 (14.42 to 15.12)
**2014**	12437	10.08 (9.90 to 10.26)	5431	7.20 (7.01 to 7.40)	7006	14.36 (14.03 to 14.70)
**2015**	12775	10.13 (9.96 to 10.31)	5491	7.14 (6.95 to 7.33)	7284	14.53 (14.19 to 14.86)
**2016**	13390	10.40 (10.23 to 10.58)	5678	7.22 (7.03 to 7.41)	7712	14.96 (14.63 to 15.30)
**2017**	14105	10.67 (10.49 to 10.85)	6018	7.56 (7.37 to 7.76)	8087	15.14 (14.81 to 15.47)
**2018**	14800	10.90 (10.72 to 11.08)	6201	7.63 (7.43 to 7.82)	8599	15.61 (15.27 to 15.94)
**2019**	15272	10.98 (10.80 to 11.15)	6284	7.58 (7.39 to 7.77)	8988	15.72 (15.40 to 16.05)
**2020**	19441	13.71 (13.51 to 13.90)	7985	9.51 (9.30 to 9.72)	11456	19.56 (19.20 to 19.92)
**2021**	17041	12.65 (12.46 to 12.84)	6959	8.84 (8.63 to 9.05)	10082	18.00 (17.64 to 18.36)
**2022**	17091	11.90 (11.72 to 12.08)	6876	8.15 (7.96 to 8.35)	10215	17.23 (16.89 to 17.57)
**2023**	15944	10.99 (10.82 to 11.16)	6414	7.64 (7.45 to 7.83)	9530	15.65 (15.34 to 15.98)
**2024**	16284	10.75 (10.59 to 10.92)	6396	7.33 (7.15 to 7.51)	9888	15.47 (15.17 to 15.78)
**2025**	16851	11.15 (10.98 to 11.32)	6389	7.34 (7.16 to 7.52)	10462	16.37 (16.06 to 16.69)

**Total/Average**	337721	10.26 (10.08 to 10.44)	146894	7.35 (7.15 to 7.55)	190827	14.69 (14.34 to 15.04)

Table S4. National Trends and Demographic Disparities in Mortality Involving Co-Recorded Parkinson's Disease and Dementia in the United States stratified by Age group, 1999–2025.

Year	45-64 Years		65-85+ Years	
	Deaths	AAMR (95% CI)	Deaths	AAMR (95% CI)
**1999**	54	0.08 (0.06 - 0.10)	5398	15.72 (15.30 - 16.14)
**2000**	93	0.16 (0.13 - 0.19)	8065	23.20 (22.69 - 23.71)
**2001**	103	0.16 (0.13 - 0.19)	8448	23.94 (23.43 - 24.45)
**2002**	88	0.12 (0.09 - 0.15)	8928	24.97 (24.45 - 25.48)
**2003**	117	0.16 (0.13 - 0.19)	9746	26.87 (26.34 - 27.40)
**2004**	132	0.16 (0.13 - 0.19)	10004	27.26 (26.72 - 27.79)
**2005**	168	0.20 (0.17 - 0.23)	10559	28.27 (27.73 - 28.81)
**2006**	140	0.16 (0.13 - 0.18)	10123	26.57 (26.06 - 27.09)
**2007**	153	0.16 (0.13 - 0.18)	10536	27.22 (26.70 - 27.74)
**2008**	138	0.16 (0.13 - 0.18)	10663	27.08 (26.56 - 27.59)
**2009**	142	0.16 (0.13 - 0.18)	10553	26.34 (25.84 - 26.85)
**2010**	151	0.16 (0.13 - 0.18)	11283	27.77 (27.26 - 28.29)
**2011**	180	0.16 (0.13 - 0.18)	11730	28.12 (27.60 - 28.63)
**2012**	191	0.16 (0.13 - 0.18)	12027	28.16 (27.65 - 28.67)
**2013**	211	0.20 (0.17 - 0.22)	12166	27.85 (27.35 - 28.35)
**2014**	182	0.16 (0.13 - 0.18)	12255	27.51 (27.02 - 28.00)
**2015**	207	0.20 (0.17 - 0.22)	12568	27.59 (27.11 - 28.08)
**2016**	219	0.26 (0.22 - 0.29)	13171	28.23 (27.75 - 28.72)
**2017**	259	0.24 (0.21 - 0.27)	13846	29.01 (28.52 - 29.50)
**2018**	220	0.20 (0.17 - 0.22)	14580	29.71 (29.23 - 30.20)
**2019**	251	0.24 (0.21 - 0.27)	15021	29.85 (29.37 - 30.33)
**2020**	336	0.31 (0.28 - 0.35)	19105	37.24 (36.71 - 37.77)
**2021**	320	0.28 (0.24 - 0.31)	16721	34.40 (33.88 - 34.93)
**2022**	311	0.34 (0.30 - 0.37)	16780	32.21 (31.72 - 32.70)
**2023**	276	0.24 (0.20 - 0.27)	15668	29.88 (29.41 - 30.35)
**2024**	276	0.24 (0.20 - 0.27)	16008	29.24 (28.78 - 29.69)
**2025**	269	0.24 (0.20 - 0.27)	16582	30.33 (29.87 - 30.80)
**Total/Average**	5187	0.20 (0.16 - 0.22)	332534	27.95 (27.45 - 28.45)

Table S5. National Trends and Demographic Disparities in Mortality Involving Co-Recorded Parkinson's Disease and Dementia in the United States stratified by Race, 1999–2025.

Year	NH Asian or Pacific Islander		NH Black or African American		NH White		Hispanic or Latino	
	Deaths	AAMR (95% CI)	Deaths	AAMR (95% CI)	Deaths	AAMR (95% CI)	Deaths	AAMR (95% CI)
**1999**	31	1.89 (1.27 to 2.70)	220	3.06 (2.65 to 3.46)	5043	6.19 (6.02 to 6.36)	152	4.15 (3.48 to 4.82)
**2000**	77	4.32 (3.39 to 5.42)	325	4.50 (4.01 to 4.99)	7542	9.14 (8.93 to 9.34)	205	5.30 (4.57 to 6.04)
**2001**	86	4.33 (3.45 to 5.37)	347	4.71 (4.21 to 5.20)	7851	9.39 (9.18 to 9.60)	242	5.72 (4.98 to 6.45)
**2002**	114	5.38 (4.38 to 6.39)	383	5.21 (4.69 to 5.74)	8255	9.76 (9.55 to 9.97)	240	5.51 (4.81 to 6.22)
**2003**	121	5.33 (4.36 to 6.29)	423	5.62 (5.08 to 6.16)	9008	10.56 (10.34 to 10.77)	285	6.26 (5.53 to 7.00)
**2004**	145	6.04 (5.05 to 7.04)	420	5.52 (4.99 to 6.05)	9218	10.69 (10.47 to 10.91)	333	6.82 (6.08 to 7.57)
**2005**	147	5.55 (4.64 to 6.46)	468	5.97 (5.43 to 6.52)	9729	11.12 (10.90 to 11.34)	361	6.94 (6.21 to 7.67)
**2006**	187	6.79 (5.81 to 7.77)	485	6.14 (5.59 to 6.69)	9159	10.34 (10.13 to 10.56)	406	7.46 (6.73 to 8.19)
**2007**	174	5.93 (5.04 to 6.82)	496	6.11 (5.57 to 6.66)	9601	10.69 (10.47 to 10.90)	390	6.73 (6.06 to 7.41)
**2008**	147	4.69 (3.93 to 5.45)	479	5.72 (5.20 to 6.24)	9692	10.65 (10.44 to 10.87)	455	7.55 (6.85 to 8.25)

**2009**	180	5.42 (4.62 to 6.22)	478	5.66 (5.15 to 6.17)	9544	10.37 (10.16 to 10.58)	455	7.15 (6.49 to 7.81)
**2010**	194	5.53 (4.74 to 6.31)	482	5.52 (5.03 to 6.02)	10204	10.96 (10.75 to 11.17)	520	7.82 (7.14 to 8.50)
**2011**	224	5.84 (5.07 to 6.61)	531	5.87 (5.37 to 6.38)	10573	11.15 (10.94 to 11.37)	551	7.61 (6.97 to 8.25)
**2012**	222	5.44 (4.72 to 6.16)	598	6.38 (5.87 to 6.90)	10776	11.17 (10.96 to 11.39)	588	7.71 (7.08 to 8.33)
**2013**	221	5.01 (4.34 to 5.67)	599	6.16 (5.66 to 6.66)	10867	11.07 (10.86 to 11.28)	661	8.02 (7.40 to 8.63)
**2014**	262	5.48 (4.81 to 6.14)	571	5.72 (5.24 to 6.19)	10884	10.97 (10.76 to 11.18)	682	7.78 (7.19 to 8.37)
**2015**	284	5.47 (4.83 to 6.11)	639	6.13 (5.65 to 6.61)	11054	10.98 (10.78 to 11.19)	754	8.08 (7.50 to 8.66)
**2016**	301	5.46 (4.84 to 6.08)	670	6.26 (5.78 to 6.74)	11589	11.33 (11.12 to 11.54)	786	7.98 (7.42 to 8.55)
**2017**	367	6.25 (5.61 to 6.90)	723	6.45 (5.97 to 6.93)	12196	11.70 (11.49 to 11.91)	762	7.42 (6.89 to 7.95)
**2018**	362	5.85 (5.24 to 6.46)	795	6.90 (6.41 to 7.38)	12727	11.93 (11.72 to 12.14)	863	7.96 (7.42 to 8.49)
**2019**	412	6.25 (5.64 to 6.86)	756	6.28 (5.82 to 6.73)	13127	12.09 (11.89 to 12.30)	920	8.09 (7.56 to 8.61)
**2020**	474	6.78 (6.16 to 7.39)	1063	8.49 (7.97 to 9.01)	16548	15.06 (14.83 to 15.29)	1294	10.92 (10.32 to 11.52)
**2021**	473	7.04 (6.42 to 7.71)	934	7.82 (7.32 to 8.35)	14537	14.01 (13.78 to 14.24)	1043	8.99 (8.44 to 9.56)
**2022**	447	6.13 (5.57 to 6.73)	877	6.97 (6.51 to 7.45)	14634	13.21 (13.00 to 13.43)	1072	8.78 (8.26 to 9.33)
**2023**	466	6.10 (5.55 to 6.68)	868	6.65 (6.21 to 7.12)	13551	12.20 (12.00 to 12.41)	1018	8.02 (7.53 to 8.53)
**2024**	427	5.14 (4.66 to 5.65)	892	6.56 (6.13 to 7.01)	13897	12.16 (11.96 to 12.37)	1023	7.48 (7.02 to 7.95)
**2025**	482	5.84 (5.32 to 6.38)	912	6.75 (6.31 to 7.21)	14329	12.50 (12.29 to 12.71)	1077	7.84 (7.37 to 8.33)
**Total/Average**	7027	5.53 (4.79 to 6.29)	16434	6.04 (5.55 to 6.54)	296135	11.16 (10.95 to 11.37)	17138	7.41 (6.79 to 8.04)

Table S6. National Trends and Demographic Disparities in Mortality Involving Co-Recorded Parkinson's Disease and Dementia in the United States stratified by Urbanization, 1999–2020.

**Year**	**Metropolitan**		**Non-metropolitan**	
	**Deaths**	**AAMR (95% CI)**	**Deaths**	**AAMR (95% CI)**
**1999**	4416	5.81 (5.64 to 5.98)	1036	5.62 (5.27 to 5.96)
**2000**	6585	8.53 (8.33 to 8.74)	1573	8.46 (8.04 to 8.88)
**2001**	6930	8.8 (8.59 to 9.01)	1621	8.68 (8.26 to 9.1)
**2002**	7263	9.1 (8.89 to 9.31)	1753	9.32 (8.88 to 9.75)
**2003**	7999	9.84 (9.62 to 10.05)	1864	9.85 (9.4 to 10.29)
**2004**	8268	10.02 (9.8 to 10.23)	1868	9.85 (9.4 to 10.3)
**2005**	8700	10.33 (10.12 to 10.55)	2027	10.57 (10.11 to 11.03)
**2006**	8368	9.73 (9.53 to 9.94)	1895	9.75 (9.31 to 10.19)
**2007**	8646	9.88 (9.68 to 10.09)	2043	10.39 (9.94 to 10.84)
**2008**	8732	9.81 (9.6 to 10.02)	2069	10.44 (9.99 to 10.89)
**2009**	8628	9.51 (9.31 to 9.71)	2067	10.33 (9.88 to 10.78)
**2010**	9309	10.11 (9.91 to 10.32)	2125	10.51 (10.06 to 10.95)
**2011**	9695	10.2 (10 to 10.41)	2215	10.75 (10.3 to 11.2)
**2012**	9923	10.2 (10 to 10.4)	2295	10.98 (10.53 to 11.43)
**2013**	10134	10.15 (9.95 to 10.35)	2243	10.52 (10.09 to 10.96)
**2014**	10190	9.99 (9.8 to 10.19)	2247	10.47 (10.04 to 10.91)
**2015**	10448	10.01 (9.81 to 10.2)	2327	10.64 (10.2 to 11.07)
**2016**	10994	10.31 (10.11 to 10.5)	2396	10.81 (10.37 to 11.25)

**2017**	11370	10.38 (10.19 to 10.57)	2735	12.03 (11.57 to 12.48)
**2018**	11965	10.62 (10.43 to 10.81)	2835	12.3 (11.84 to 12.75)
**2019**	12377	10.68 (10.49 to 10.87)	2895	12.36 (11.91 to 12.81)
**2020**	15793	13.35 (13.14 to 13.56)	3648	15.29 (14.79 to 15.79)
**Total/Average**	206733	9.88 (9.68 to 10.08)	47777	10.45 (10.01 to 10.89)

Table S7. National Trends and Demographic Disparities in Mortality Involving Co-Recorded Parkinson's Disease and Dementia in the United States stratified by Census region, 1999–2025.

Year	Northeast		Midwest		South		West	
	Deaths	AAMR (95% CI)	Deaths	AAMR (95% CI)	Deaths	AAMR (95% CI)	Deaths	AAMR (95% CI)
**1999**	1103	5.39 (5.07 to 5.7)	1411	6.12 (5.8 to 6.44)	1954	5.96 (5.69 to 6.22)	984	5.35 (5.01 to 5.68)
**2000**	1641	7.87 (7.49 to 8.25)	2200	9.44 (9.04 to 9.83)	2611	7.85 (7.55 to 8.15)	1706	9.14 (8.7 to 9.57)
**2001**	1763	8.36 (7.97 to 8.75)	2329	9.89 (9.49 to 10.3)	2671	7.91 (7.61 to 8.21)	1788	9.31 (8.87 to 9.74)
**2002**	1769	8.28 (7.89 to 8.66)	2501	10.51 (10.1 to 10.92)	2853	8.39 (8.08 to 8.7)	1893	9.66 (9.22 to 10.1)
**2003**	1866	8.65 (8.25 to 9.04)	2634	10.99 (10.57 to 11.41)	3278	9.49 (9.16 to 9.81)	2085	10.4 (9.95 to 10.85)
**2004**	1919	8.82 (8.43 to 9.22)	2762	11.35 (10.93 to 11.77)	3284	9.39 (9.07 to 9.71)	2171	10.63 (10.18 to 11.08)
**2005**	2002	9.08 (8.68 to 9.48)	2974	12.1 (11.66 to 12.53)	3507	9.78 (9.45 to 10.1)	2244	10.67 (10.23 to 11.11)
**2006**	1901	8.53 (8.15 to 8.92)	2765	11.1 (10.68 to 11.51)	3370	9.23 (8.92 to 9.54)	2227	10.36 (9.93 to 10.79)
**2007**	2085	9.25 (8.85 to 9.65)	2780	11 (10.59 to 11.41)	3600	9.65 (9.34 to 9.97)	2224	10.1 (9.68 to 10.52)
**2008**	2009	8.79 (8.41 to 9.18)	2891	11.33 (10.92 to 11.75)	3534	9.27 (8.96 to 9.58)	2367	10.54 (10.11 to 10.97)
**2009**	1990	8.6 (8.22 to 8.99)	2789	10.83 (10.43 to 11.24)	3589	9.24 (8.93 to 9.54)	2327	10.04 (9.63 to 10.45)
**2010**	2161	9.32 (8.92 to 9.71)	2862	10.93 (10.52 to 11.33)	3879	9.8 (9.49 to 10.11)	2532	10.73 (10.31 to 11.15)
**2011**	2264	9.57 (9.17 to 9.97)	3046	11.49 (11.08 to 11.9)	4032	9.83 (9.53 to 10.14)	2568	10.5 (10.09 to 10.91)
**2012**	2194	9.1 (8.72 to 9.49)	3093	11.47 (11.06 to 11.88)	4215	10.05 (9.75 to 10.36)	2716	10.78 (10.38 to 11.19)
**2013**	2329	9.62 (9.23 to 10.02)	3144	11.46 (11.05 to 11.86)	4256	9.8 (9.51 to 10.1)	2648	10.23 (9.84 to 10.62)
**2014**	2299	9.37 (8.98 to 9.76)	3320	12 (11.59 to 12.42)	4181	9.38 (9.1 to 9.67)	2637	9.9 (9.52 to 10.28)
**2015**	2338	9.44 (9.05 to 9.83)	3254	11.58 (11.17 to 11.98)	4419	9.63 (9.34 to 9.92)	2764	10.05 (9.67 to 10.43)
**2016**	2355	9.38 (8.99 to 9.76)	3391	11.95 (11.55 to 12.36)	4722	9.98 (9.69 to 10.26)	2922	10.36 (9.99 to 10.74)
**2017**	2541	9.83 (9.45 to 10.22)	3450	11.86 (11.46 to 12.26)	5148	10.6 (10.31 to 10.9)	2966	10.27 (9.9 to 10.65)
**2018**	2626	10.05 (9.66 to 10.44)	3731	12.6 (12.19 to 13)	5414	10.76 (10.47 to 11.05)	3029	10.16 (9.8 to 10.53)
**2019**	2625	9.89 (9.51 to 10.28)	3755	12.43 (12.03 to 12.83)	5703	10.98 (10.69 to 11.26)	3189	10.4 (10.04 to 10.76)
**2020**	3412	12.73 (12.3 to 13.17)	4816	15.88 (15.43 to 16.33)	7448	13.98 (13.66 to 14.3)	3765	11.94 (11.55 to 12.32)
**2021**	2806	10.92 (10.51 to 11.33)	3867	13.45 (13.03 to 13.89)	6790	13.47 (13.15 to 13.8)	3578	12.09 (11.7 to 12.5)
**2022**	2712	9.96 (9.58 to 10.34)	3927	12.86 (12.45 to 13.27)	6960	12.86 (12.56 to 13.17)	3492	10.94 (10.58 to 11.31)
**2023**	2476	9.05 (8.69 to 9.42)	3685	12.04 (11.65 to 12.44)	6655	12.17 (11.88 to 12.47)	3128	9.69 (9.36 to 10.04)
**2024**	2578	9.14 (8.79 to 9.5)	3796	11.94 (11.56 to 12.33)	6786	11.85 (11.57 to 12.14)	3124	9.22 (8.89 to 9.55)
**2025**	2612	9.23 (8.88 to 9.59)	3992	12.54 (12.15 to 12.94)	7022	12.29 (12.01 to 12.59)	3225	9.54 (9.22 to 9.88)

**Total/Average**	60376	9.19 (8.81 to 9.58)	85165	11.52 (11.12 to 11.93)	121881	10.13 (9.83 to 10.44)	70299	10.11 (9.72 to 10.51)

Table S8. National Trends and Demographic Disparities in Mortality Involving Co-Recorded Parkinson's Disease and Dementia in the United States stratified by State, 1999–2025.

State	1999-2020		2021-2025		1999-2025
	Deaths	AAMR (95% CI)	Deaths	AAMR (95% CI)	Deaths
**Alabama**	3135	8.1 (7.81 to 8.38)	868	7.69 (7.19 - 8.23)	4003
**Alaska**	320	11.64 (10.34 to 12.94)	100	9.84 (7.96 - 12.06)	420
**Arizona**	3803	7.21 (6.98 to 7.44)	1439	7.98 (7.57 - 8.41)	5242
**Arkansas**	2077	8.29 (7.93 to 8.65)	771	11.39 (10.6 - 12.24)	2848
**California**	26773	9.89 (9.77 to 10.01)	6801	8.68 (8.47 - 8.89)	33574
**Colorado**	3939	11.96 (11.59 to 12.34)	1737	16.07 (15.32 - 16.86)	5676
**Connecticut**	3348	9.78 (9.45 to 10.12)	804	9.15 (8.53 - 9.81)	4152
**Delaware**	742	9.68 (8.98 to 10.37)	356	13.99 (12.56 - 15.57)	1098
**District of Columbia**	280	6.17 (5.44 to 6.9)	85	7.43 (5.93 - 9.23)	365
**Florida**	15693	7.48 (7.37 to 7.6)	6867	10.56 (10.31 - 10.81)	22560
**Georgia**	5115	8.62 (8.38 to 8.86)	1990	10.08 (9.64 - 10.54)	7105
**Hawaii**	1056	8.09 (7.6 to 8.58)	219	5.73 (4.99 - 6.59)	1275
**Idaho**	1290	11.07 (10.47 to 11.68)	509	13.06 (11.94 - 14.26)	1799
**Illinois**	10005	9.64 (9.45 to 9.83)	2432	8.85 (8.5 - 9.21)	12437
**Indiana**	6082	11.61 (11.32 to 11.91)	1855	12.97 (12.38 - 13.58)	7937
**Iowa**	3780	12.26 (11.87 to 12.66)	1138	15.15 (14.28 - 16.07)	4918
**Kansas**	2705	10.75 (10.35 to 11.16)	796	12.48 (11.62 - 13.39)	3501
**Kentucky**	4219	12.45 (12.07 to 12.83)	1945	20.42 (19.52 - 21.36)	6164
**Louisiana**	2474	7.32 (7.03 to 7.61)	896	9.54 (8.92 - 10.19)	3370
**Maine**	1590	12.11 (11.51 to 12.71)	463	12.13 (11.04 - 13.31)	2053
**Maryland**	5108	11.63 (11.31 to 11.95)	1988	15.2 (14.53 - 15.88)	7096
**Massachusetts**	5026	8.27 (8.04 to 8.5)	1713	10.64 (10.14 - 11.16)	6739
**Michigan**	8831	10.37 (10.15 to 10.58)	2575	11.2 (10.76 - 11.64)	11406
**Minnesota**	6970	15.5 (15.13 to 15.87)	2545	20.07 (19.29 - 20.87)	9515
**Mississippi**	2123	9.42 (9.02 to 9.82)	828	13.41 (12.51 - 14.37)	2951
**Missouri**	5050	9.64 (9.37 to 9.9)	1645	11.8 (11.24 - 12.39)	6695
**Montana**	847	9.35 (8.72 to 9.99)	250	9.31 (8.18 - 10.58)	1097
**Nebraska**	2267	13.72 (13.15 to 14.29)	758	17.98 (16.72 - 19.33)	3025
**Nevada**	1141	6.58 (6.2 to 6.97)	595	9.36 (8.62 - 10.16)	1736
**New Hampshire**	1364	12.19 (11.54 to 12.84)	462	13.41 (12.21 - 14.71)	1826
**New Jersey**	6419	8.32 (8.11 to 8.52)	1575	7.54 (7.17 - 7.92)	7994
**New Mexico**	1537	9.71 (9.22 to 10.2)	406	8.14 (7.36 - 8.99)	1943
**New York**	13743	8.02 (7.88 to 8.15)	4006	8.56 (8.3 - 8.83)	17749
**North Carolina**	7805	10.77 (10.53 to 11.01)	2695	11.95 (11.5 - 12.41)	10500
**North Dakota**	885	13.09 (12.21 to 13.97)	243	14.05 (12.32 - 16)	1128
**Ohio**	12312	12.01 (11.8 to 12.22)	3163	11.8 (11.39 - 12.22)	15475

**Oklahoma**	3319	10.89 (10.51 to 11.26)	1299	16.06 (15.2 - 16.97)	4618
**Oregon**	4375	13.02 (12.63 to 13.41)	1656	16.91 (16.1 - 17.76)	6031
**Pennsylvania**	13640	10.41 (10.23 to 10.58)	3502	10.72 (10.36 - 11.08)	17142
**Rhode Island**	1231	11.49 (10.84 to 12.14)	411	15.58 (14.1 - 17.19)	1642
**South Carolina**	4161	11.55 (11.2 to 11.9)	2014	16.71 (15.98 - 17.47)	6175
**South Dakota**	869	11.06 (10.32 to 11.8)	267	13.4 (11.82 - 15.15)	1136
**Tennessee**	5926	11.89 (11.59 to 12.2)	2166	14.76 (14.14 - 15.4)	8092
**Texas**	18200	11.78 (11.61 to 11.95)	6635	13.61 (13.28 - 13.94)	24835
**Utah**	1547	10.4 (9.88 to 10.92)	498	10.29 (9.4 - 11.25)	2045
**Vermont**	831	14.61 (13.61 to 15.61)	248	14.8 (13 - 16.82)	1079
**Virginia**	5314	9.13 (8.88 to 9.37)	2183	12.02 (11.52 - 12.54)	7497
**Washington**	6810	13.28 (12.96 to 13.59)	2215	14.09 (13.51 - 14.7)	9025
**West Virginia**	1977	11.17 (10.68 to 11.67)	627	13.75 (12.69 - 14.89)	2604
**Wisconsin**	6142	12.05 (11.75 to 12.36)	1850	13.71 (13.09 - 14.36)	7992
**Wyoming**	314	7.51 (6.68 to 8.35)	122	9.75 (8.08 - 11.71)	436

Table S9. Percentage distribution of place of death in the United States (1999–2025), Co-Recorded Parkinson's Disease and Dementia related mortality highlighted.

Place of Death	Deaths	% Total
**Medical Facility - Inpatient**	42,206	12.50%
**Medical Facility - Outpatient or ER**	5,364	1.59%
**Medical Facility - Dead on Arrival**	478	0.14%
**Medical Facility - Status unknown**	77	0.02%
**Decedent's home**	78,578	23.26%
**Hospice facility**	18,224	5.40%
**Nursing home/long term care**	172,159	50.99%
**Other**	20,103	5.95%
**Place of death unknown**	528	0.16%
**Total**	337717	100%

Table S10. National Trends and Demographic Disparities in Mortality Involving Co-Recorded Parkinson's Disease and Dementia in the United States stratified by overall excluding 2025 provisional data, 1999–2024.

Year	COVID	
	Deaths	AAMR (95% CI)
**1999**	5452	5.75 (5.6–5.9)
**2000**	8158	8.51 (8.33–8.7)
**2001**	8551	8.78 (8.6–8.97)
**2002**	9016	9.13 (8.94–9.32)
**2003**	9863	9.85 (9.65–10.04)
**2004**	10136	9.99 (9.79–10.18)
**2005**	10727	10.38 (10.18–10.57)
**2006**	10263	9.74 (9.55–9.93)
**2007**	10689	9.97 (9.78–10.16)
**2008**	10801	9.92 (9.73–10.11)
**2009**	10695	9.65 (9.47–9.84)
**2010**	11434	10.17 (9.99–10.36)
**2011**	11910	10.3 (10.11–10.48)
**2012**	12218	10.31 (10.13–10.5)
**2013**	12377	10.23 (10.04–10.41)
**2014**	12437	10.08 (9.9–10.26)
**2015**	12775	10.13 (9.96–10.31)
**2016**	13390	10.4 (10.23–10.58)

**2017**	14105	10.67 (10.49–10.85)
**2018**	14800	10.9 (10.72–11.08)
**2019**	15272	10.98 (10.8–11.15)
**2020**	19441	13.71 (13.51–13.9)
**2021**	17041	12.65 (12.46–12.84)
**2022**	17091	11.9 (11.72–12.08)
**2023**	15944	10.99 (10.82–11.16)
**2024**	16284	10.75 (10.59–10.92)

Table S11. National Trends and Demographic Disparities in Mortality Involving Co-Recorded Parkinson's Disease and Dementia in the United States stratified by Before COVID, 1999–2019.

**Year**	**COVID**	
	**Deaths**	**AAMR (95% CI)**
**1999**	5452	5.75 (5.6–5.9)
**2000**	8158	8.51 (8.33–8.7)
**2001**	8551	8.78 (8.6–8.97)
**2002**	9016	9.13 (8.94–9.32)
**2003**	9863	9.85 (9.65–10.04)
**2004**	10136	9.99 (9.79–10.18)
**2005**	10727	10.38 (10.18–10.57)
**2006**	10263	9.74 (9.55–9.93)
**2007**	10689	9.97 (9.78–10.16)
**2008**	10801	9.92 (9.73–10.11)
**2009**	10695	9.65 (9.47–9.84)
**2010**	11434	10.17 (9.99–10.36)
**2011**	11910	10.3 (10.11–10.48)
**2012**	12218	10.31 (10.13–10.5)
**2013**	12377	10.23 (10.04–10.41)
**2014**	12437	10.08 (9.9–10.26)
**2015**	12775	10.13 (9.96–10.31)
**2016**	13390	10.4 (10.23–10.58)
**2017**	14105	10.67 (10.49–10.85)
**2018**	14800	10.9 (10.72–11.08)
**2019**	15272	10.98 (10.8–11.15)

Table S12. National Trends and Demographic Disparities in Mortality Involving Co-Recorded Parkinson's Disease and Dementia in the United States stratified by sensitivity analysis, 1999–2025.

**Year**	**UCD Dementia**		**UCD Dementia**	
	**Deaths**	**AAMR (95% CI)**	**Deaths**	**AAMR (95% CI)**
**1999**	74099	78.7 (78.13–79.26)	14478	15.28 (15.04–15.53)
**2000**	83367	87.16 (86.57–87.75)	15549	16.21 (15.95–16.46)
**2001**	92190	94.78 (94.16–95.39)	16412	16.84 (16.58–17.1)
**2002**	101708	103.23 (102.6–103.87)	16819	17.03 (16.77–17.29)
**2003**	110237	109.93 (109.28–110.58)	17846	17.8 (17.54–18.06)
**2004**	113955	112.05 (111.39–112.7)	17864	17.61 (17.35–17.87)
**2005**	129285	124.22 (123.55–124.9)	19406	18.81 (18.54–19.07)
**2006**	151093	141.63 (140.92–142.35)	19404	18.46 (18.2–18.72)
**2007**	155752	142.55 (141.84–143.26)	19893	18.61 (18.35–18.87)
**2008**	178544	159.93 (159.19–160.68)	20289	18.65 (18.4–18.91)
**2009**	177122	155.18 (154.45–155.9)	20368	18.45 (18.19–18.7)
**2010**	196034	168.65 (167.9–169.4)	21817	19.45 (19.2–19.71)
**2011**	212543	176.91 (176.15–177.66)	22949	19.94 (19.68–20.2)
**2012**	222842	181.22 (180.46–181.98)	23618	20.03 (19.77–20.29)
**2013**	233747	185.45 (184.69–186.21)	24973	20.69 (20.43–20.95)
**2014**	239120	185.86 (185.11–186.61)	25926	21.03 (20.77–21.29)
**2015**	245074	186.44 (185.69–187.18)	27723	21.95 (21.69–22.21)
**2016**	248968	185.85 (185.11–186.58)	29438	22.86 (22.6–23.12)
**2017**	261298	191.09 (190.35–191.83)	31688	23.97 (23.71–24.24)
**2018**	266397	190.61 (189.89–191.34)	33529	24.64 (24.38–24.91)
**2019**	271378	190.75 (190.03–191.47)	35025	25.13 (24.87–25.4)
**2020**	303438	209.96 (209.21–210.71)	39957	28.1 (27.82–28.38)
**2021**	278329	208.52 (207.74–209.29)	38108	28.23 (27.94–28.51)
**2022**	291400	202.81 (202.08–203.55)	39431	27.33 (27.06–27.6)

**2023**	285269	201.2 (200.46–201.94)	39714	27.35 (27.08–27.62)
**2024**	297551	201.68 (200.96–202.41)	40414	26.71 (26.45–26.97)
**2025**	309095	209.38 (208.64–210.12)	43000	28.43 (28.17–28.71)

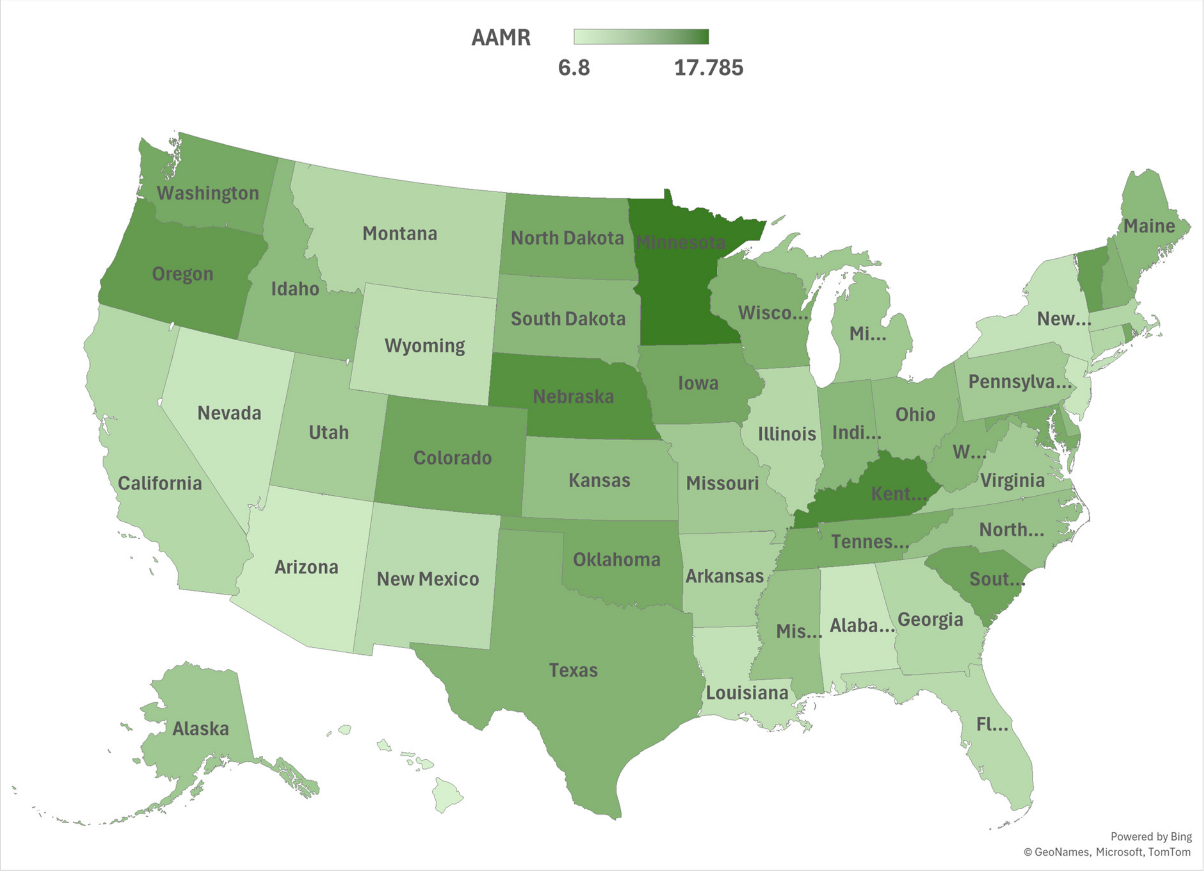

Figure S1. National Trends and Demographic Disparities in Mortality Involving Co-Recorded Parkinson's Disease and Dementia in the United States stratified by States adults aged ≥45 years, 1999–2025.

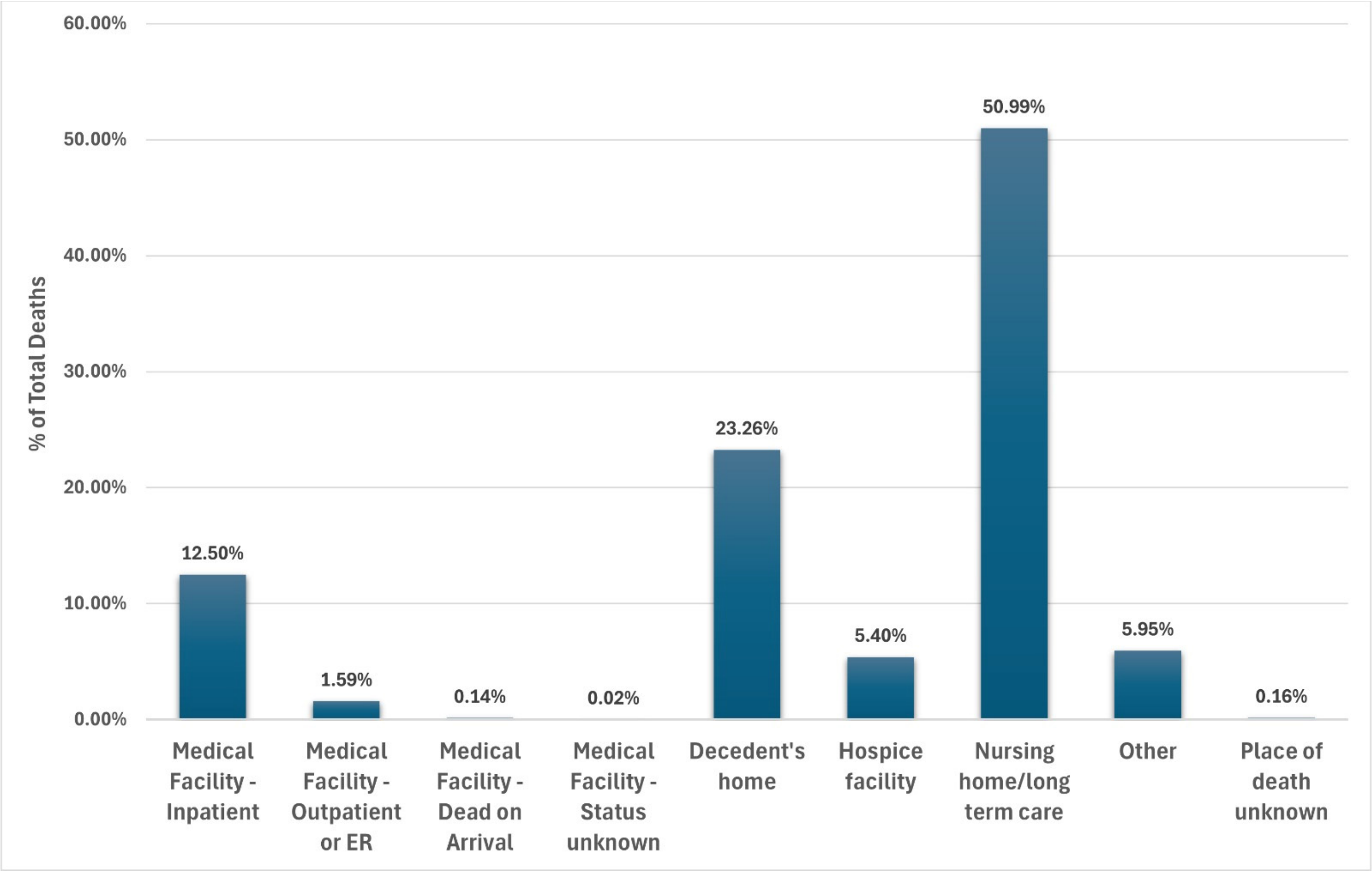

Figure S2. Place-of-death trends in the United States (1999–2025), highlighting mortality involving co-recorded Parkinson's Disease and dementia.

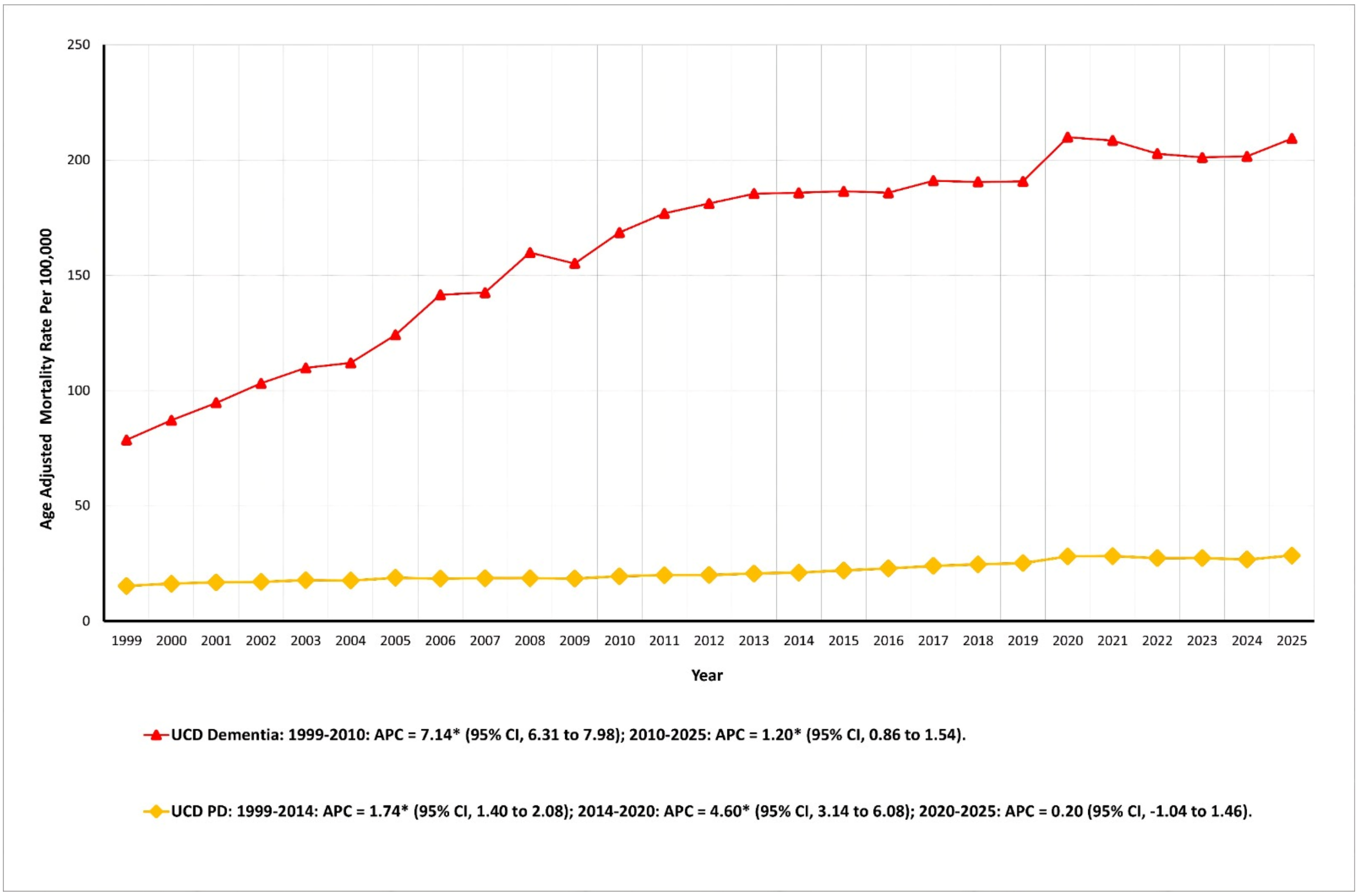

Figure S3. Sensitivity analysis stratified age-adjusted mortality rates per 100,000 for Co-Recorded Parkinson's Disease and Dementia related mortality in United States adults aged ≥45 years, 1999–2025.

https://doi.org/10.3390/xxxxx
